# Detection of Known and Novel Small Proteins in *Pseudomonas stutzeri* Using a Combination of Bottom-Up and Digest-Free Proteomics and Proteogenomics

**DOI:** 10.1021/acs.analchem.3c00676

**Published:** 2023-08-03

**Authors:** Jakob Meier-Credo, Benjamin Heiniger, Christian Schori, Fiona Rupprecht, Hartmut Michel, Christian H. Ahrens, Julian D. Langer

**Affiliations:** † Proteomics, Max Planck Institute of Biophysics, 60438 Frankfurt am Main, Germany; ‡ Molecular Ecology, Agroscope & SIB Swiss Institute of Bioinformatics, 8046 Zürich, Switzerland; § Proteomics, Max Planck Institute for Brain Research, 60438 Frankfurt am Main, Germany; ∥ Department of Molecular Membrane Biology, Max Planck Institute of Biophysics, 60438 Frankfurt am Main, Germany

## Abstract

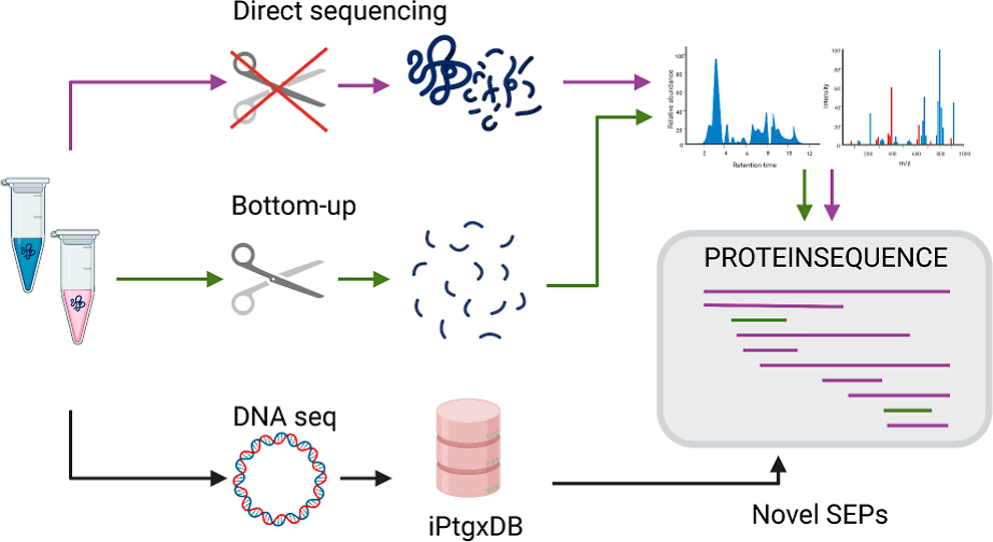

Small proteins of around 50 aa in length have been largely overlooked in genetic and biochemical assays due to the inherent challenges with detecting and characterizing them. Recent discoveries of their critical roles in many biological processes have led to an increased recognition of the importance of small proteins for basic research and as potential new drug targets. One example is CcoM, a 36 aa subunit of the *cbb3*-type oxidase that plays an essential role in adaptation to oxygen-limited conditions in *Pseudomonas stutzeri (P. stutzeri)*, a model for the clinically relevant, opportunistic pathogen *Pseudomonas aeruginosa*. However, as no comprehensive data were available in *P. stutzeri*, we devised an integrated, generic approach to study small proteins more systematically. Using the first complete genome as basis, we conducted bottom-up proteomics analyses and established a digest-free, direct-sequencing proteomics approach to study cells grown under aerobic and oxygen-limiting conditions. Finally, we also applied a proteogenomics pipeline to identify missed protein-coding genes. Overall, we identified 2921 known and 29 novel proteins, many of which were differentially regulated. Among 176 small proteins 16 were novel. Direct sequencing, featuring a specialized precursor acquisition scheme, exhibited advantages in the detection of small proteins with higher (up to 100%) sequence coverage and more spectral counts, including sequences with high proline content. Three novel small proteins, uniquely identified by direct sequencing and not conserved beyond *P. stutzeri*, were predicted to form an operon with a conserved protein and may represent *de novo* genes. These data demonstrate the power of this combined approach to study small proteins in *P. stutzeri* and show its potential for other prokaryotes.

## Introduction

In recent years, an increasing number of small proteins of around 50 amino acids in length have been discovered to play important roles in many prokaryotes and archaea.^1−3^ Amongst others, they were found to represent key elements in cellular communication, stress response, respiratory chain complexes, photosystem assembly, community adaptation, and antibiotic resistance, but had been missed previously by genetics and proteomics due to the inherent challenges with annotation and detection. Thus, there is an increasing recognition that small proteins need to be studied systematically in key model systems and biologically relevant organisms.

**Figure 1:**
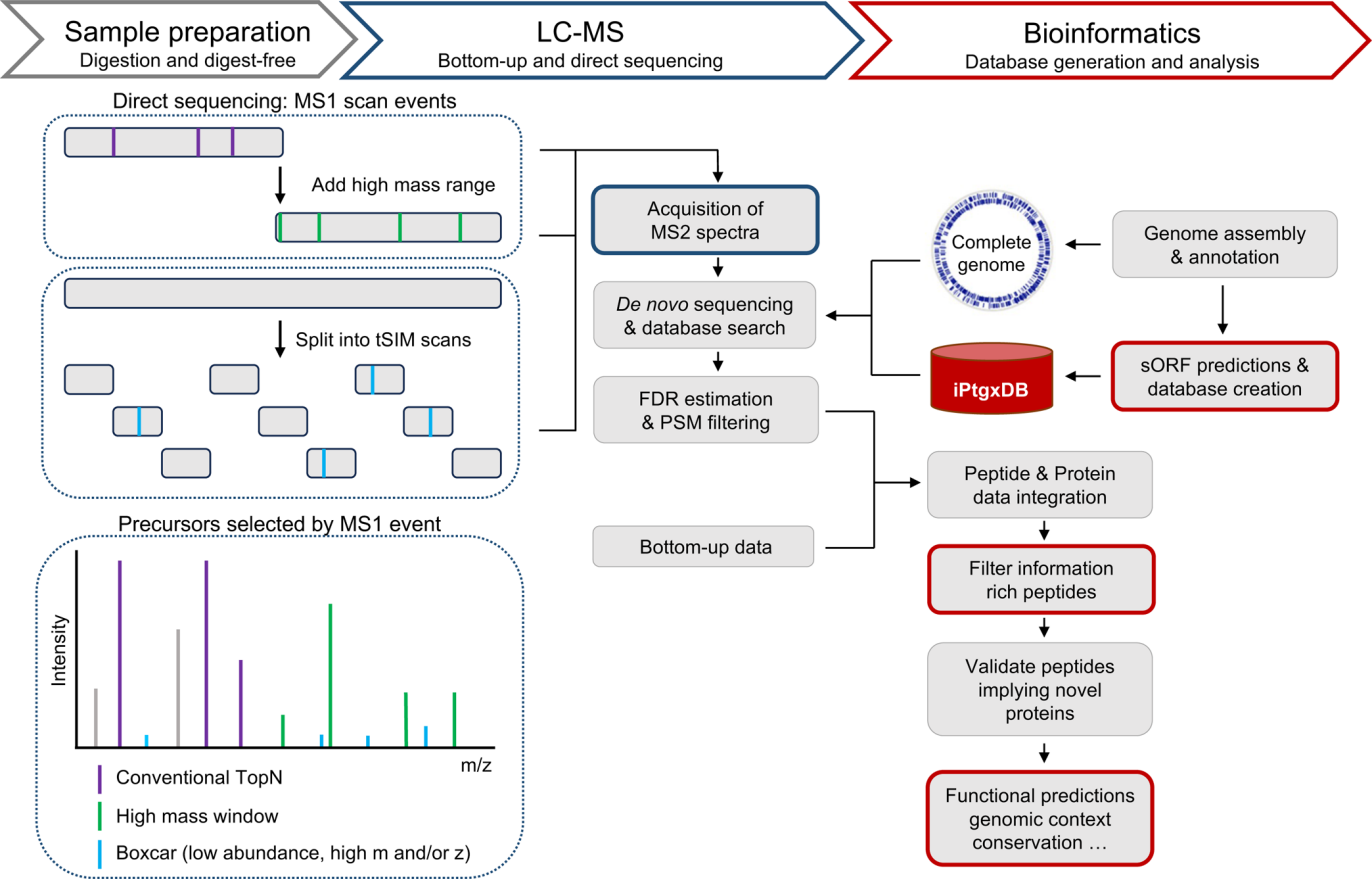
Direct-sequencing acquisition strategy and bioinformatics pipeline. To maximize small protein detection, we developed an acquisition strategy that extends conventional data-dependent acquisition methods by expanding the mass range and including low-intensity candidate ions using tSIM scans. Fragment spectra are then matched to an iPtgxDB that virtually covers the entire protein coding potential including novel CDS. Bottom-up and direct-sequencing data are merged and then curated using PeptideClassifier^17^ to focus on information-rich peptides, and further analyzed based on genomic information, proteomic evidence, and computational predictions.

*Pseudomonas* comprise a ubiquitous genus of Gram-negative bacteria with high metabolic diversity.^4^*Pseudomonas* species have adapted to and colonize a wide range of environmental niches including marine, freshwater, and terrestrial environments, and also form intimate associations with plants and animals.^5^*Pseudomonas aeruginosa*, the type species from Pseudomonas, is one of the most widely spread, opportunistic human pathogens (∼10% of nosocomial infections)^6^ and carbapenem-resistant strains were recently classified in the highest priority group of “critical pathogens” by the WHO (2017). *Pseudomonas stutzeri*, another wide-spread species^7^ and close relative of *P. aeruginosa*, has been used as a model system due to its high homology and similar metabolism.^8,9^ One key metabolic trait is its ability to proliferate in conditions of partial or total oxygen depletion, using nitrate or nitrite as terminal electron acceptors. This ability is thought to play a critical role in pathogenicity, as oxygen diffusion can be reduced during lung infections by thick layers of lung mucus or bacteria-produced fluids. The denitrification cascade has been studied and characterized in great detail in these and other bacteria, but only a few studies have been conducted on the overall metabolic changes and proteome remodeling under oxygen-limited conditions.^10^ While important roles in metabolic adaptation to different environments^9^ and for community metabolism^11^ have been described for small proteins, their comprehensive detection and analysis by bottom-up or shotgun proteomics, which is still the predominant workflow to profile proteomes, is challenging.^12,13^ Almost all MS-based methods rely on genome sequence annotation as the basis for protein search databases, and thus greatly benefit from complete genome sequences, which are often not available for specialized organisms. However, the comprehensive and accurate annotation of genome sequences is still an unsolved issue. Variable length thresholds (between 50 and 100 aa) have been applied to limit the inclusion of spurious short ORFs that do not encode functional proteins. Experimental approaches that can provide direct evidence for translated mRNAs like ribosome profiling^14^ (Ribo-seq) and stable proteins like MS-based proteogenomics have a great potential to improve genome annotations. Yet, Ribo-seq needs to be adapted for each bacterial species,^15^ while adaptations to the standard shotgun proteomics workflow are needed to detect small proteins, which are often only identified by a single or a few peptides. In fact, a very recent study showed that the two technologies deliver complementary data in terms of novel small protein identifications.^15^ Top-down proteomics is developing into a promising tool to fill this detection gap, with several studies recently reporting both known and novel, previously undetected small proteins and proteoforms in different organisms.^13,16^ Here, we adjust and extend this approach by exploiting non-specific cleavage during sample preparation and analysis: Our digest-free “direct sequencing” method combines a modified “top-down” approach with a search against a custom proteogenomics database that includes both annotated proteins and many potentially novel open reading frame-encoded proteoforms (Figure 1). In this pipeline, we optimized sample preparation for small protein extraction and modified data acquisition parameters to unify and maximize detection of full-length small proteins together with peptide fragments. *De novo* sequencing and unspecific (i.e., no enzyme) database searches then enable identification of novel small proteins and provide significantly higher sequence coverage and redundancy for the identified small proteins.

Here we performed such a comprehensive genomic and proteomics analysis of *P. stutzeri* to study proteome remodeling under aerobic and oxygen-limiting conditions. Using the first complete genome of strain ATCC 14405 as optimal basis, we identified and quantified more than 2900 proteins (over 70% of the theoretical proteome), including 160 annotated small proteins below 100 amino acids. Using our proteogenomics pipeline, we identified 29 novel, previously non-annotated proteins. While identifying a third of the proteins compared to shotgun proteomics overall, we show the particular value of direct sequencing for a more comprehensive discovery of novel small proteins.

## Methods

### Cell Culture and gDNA Extraction

*P. stutzeri* strain ATCC 14405 (=CCUG 16156) is a gamma-proteobacterium that was originally isolated from a marine environment, which has served as a model organism for denitrification studies.^18^ Recent phylogenomic analyses of the genus *Pseudomonas* suggested to split it into several genera, including *Stutzerimonas* with *Stutzerimonas stutzeri* as type species.^19^ Cell growth: *P. stutzeri* cells were essentially grown as described,^20^ cells were harvested at an OD_600_ nm of 1.6, flash-frozen in liquid nitrogen, and stored at −80 °C (further details including description of aerobic and oxygen limited conditions in Supplemental Methods). DNA extraction: Genomic DNA was extracted and purified using a QIAGEN Genomic-tip 20/G kit (Qiagen) according to the manufacturer’s instructions.

### Cell Lysis, Protein Solubilization, and Tryptic Digestion

In brief, cell pellets were homogenized using a rod sonifier (Branson) and cell lysates were reduced, alkylated, and digested using trypsin in a modified S-Trap mini protocol (Protifi). Resulting peptides were eluted and desalted with C18-SPE cartridges (Biotage). Eluates were dried in a SpeedVac.

### Small Protein Extraction

Cell lysates were extracted using UA20 buffer (20% ACN, 6 M Urea) in an ultrasonic bath. Extracts were loaded onto Microcon 30 molecular weight cutoff filter units (Merck), and the flow-throughs were collected, desalted, and purified with C18-SPE cartridges (Biotage). Eluates were dried in a SpeedVac.

### Genome Assembly and Comparison

As no complete *P. stutzeri* ATCC14405 genome was available at the National Center of Biotechnology Information’s (NCBI) non-redundant RefSeq database (Sept. 2018), the strain was sequenced and *de novo* assembled using long reads from Pacific Biosciences’ (PacBio) SMRT-RSII system (1 SMRT cell; P6-C4 chemistry; BluePippin size selected inserts > 10 kbp) and short Illumina reads (2 × 300 bp paired ends) as described.^21^ Illumina reads were used to polish the genome, to remove potential homopolymer errors and to explore the presence of plasmids.^21^ The *P. stutzeri* genome assembly based on Roche 454 GS-FLX data (RefSeq GCF_000237885.1) was compared by aligning the 130 contigs to the single chromosome in our *de novo* assembly (Table S1). Genes partially or completely missing in the short read-based assembly were visualized along with the mapped contigs (Circos v0.69-8).^22^ For more detail, see Supporting Information.

### Generation of Protein Search Databases

MS/MS data were first searched against the RefSeq annotation (4096 proteins; Feb. 18, 2019). In addition, an integrated proteogenomics search database (iPtgxDB) was created for *P. stutzeri* ATCC 14405 as described^17,23^ using the annotation from NCBI’s Prokaryotic Genome Annotation Pipeline (PGAP) as anchor annotation.^24^ A*b initio* gene predictions from Prodigal^25^ (v.2.6.3) and ChemGenome^26^ (v.2.1; with parameters: method: Swissprot space; length threshold: 70 nt; initiation codons: ATG, CTG, TTG, and GTG), and a modified in silico ORF prediction that also considers the three most frequent alternative start codons (TTG, GTG, and CTG)^27^ and sORFs down to 18 aa in length were hierarchically integrated. The different genome annotations were collapsed into annotation clusters with the same stop codon, but different start sites to consider potential longer or shorter proteoforms detectable by tryptic peptides and to achieve minimal redundancy of protein sequences (Table S2). Files (FASTA, GFF) can be downloaded (https://iptgxdb.expasy.org) and used for searches and for integrated visualization of annotated features in the context of experimental evidence.

### Mass Spectrometry

LC–MS measurements were carried out on an Ultimate 3000 nanoRSLC (Thermo Fisher) system coupled to an Orbitrap Fusion Lumos mass spectrometer (Thermo Fisher). Dried samples were redissolved in 40 μL sample buffer (95% water, 5% ACN, supplemented with 0.1% FA). Bottom-up proteomics samples were eluted in stepped gradients and analyzed in data-dependent mode, with precursors selected based on intensity, charge state, and isotope pattern. Selected precursors were isolated, subjected to HCD fragmentation, and fragment spectra were recorded in the Orbitrap. Direct-sequencing samples were eluted in stepped gradients. Peptides and proteins eluting from the column were analyzed by two methods in data-dependent mode employing split MS1 windows for precursor selection and a modified BoxCar method.^28^ Both methods used HCD fragmentation; fragment spectra were recorded in the Orbitrap. A detailed description of all LC–MS method parameters is available in the Supplementary Methods.

### Analysis of Proteomic Datasets

Mass spectrometry data files were processed with PEAKS Studio (version 10.6). *De novo* sequencing and consecutive database searches against the RefSeq and iPtgxDB databases were performed with distinct parameter sets and resulting database matches were filtered with a 0.1% peptide spectrum match (PSM) level FDR for bottom-up and 0.3% for direct sequencing to achieve an estimated protein level FDR below 1%. To increase stringency, only proteins with at least one PSM in at least 2/3 samples per growth condition were retained.

Label-free quantifications were performed with PEAKS Studio requiring at least one unique peptide per protein in 2/3 samples for both conditions. Significant fold changes were determined using PEAKSQ with an adjusted Benjamini–Hochberg FDR of 1%. We further extracted proteins detected exclusively in one condition manually by spectral counting and list them separately, with their respective PSM counts. Functional protein annotations were obtained from eggNOG-mapper.^29^

### Analysis of Novel CDS Identified by Proteogenomics

All MS/MS data were searched against the iPtgxDB with PEAKS Studio. A prediction resource-specific filter was applied on top of the stringent PSM FDR cutoffs to require more PSM evidence for novel CDS implied by ab initio (Prodigal, Chemgenome; 3 PSMs) and in silico predictions (4 PSMs), as described.^21^ The ambiguity of all peptides implying novel proteins was assessed with PeptideClassifier^17^ extended for prokaryotic proteogenomics.^23^ Their PSMs were manually evaluated to filter false-positives and create a high confidence list of 16 novel small proteins (Table S5). An additional 13 novel proteins were longer than 100 aa (Table S6).

Functional predictions for annotated and novel CDS were obtained from eggNOG-mapper^29^ (v2.1.9). Signal peptides and transmembrane domains were computed using InterProScan^30^ (v5.59-91) with the integrated software tools SignalP (v4.1), Phobius (v1.01), and TMHMM (v2.0c). Subcellular localization was predicted with PSORTb (https://www.psort.org/psortb/, v.3.0.2) and potential lipoproteins with LipoP. Additional functional and genomic context predictions were made with the Phyre2,^31^ and Operon-Mapper^32^ web servers, respectively. Operon predictions were obtained using a combined GFF file of RefSeq 2022 annotated CDS (see below) and the novel ORFs. The conservation of novel proteins was assessed by a tBlastN 2.12.0+ search against the NCBI nt database (November 2, 2022) with default parameters, except for using genetic code 11 and allowing 1,00,000 target sequences. For proteins without any hit, the search was repeated with the low complexity filter turned off. BLAST hits with at least 60% sequence coverage and 40% identity (e-value below 0.01) were summarized for different taxonomic groups (ete3 Python package) (see Tables S5, and S6). Finally, the novel proteins were compared to the latest RefSeq annotation (Aug. 1, 2022) and categorized as exact matches (identical start and stop coordinates) and stop matches (only identical stop coordinate) (Table S3).

### Additional Bioinformatic Data Analyses

An extensive master table was compiled that integrates genomic information, proteomic evidence, and computational predictions allowing researchers to filter and identify all data sets described (see Table S3). Physico-chemical parameters were calculated and biases of proteins identified by bottom-up and direct sequencing were visualized to show benefits of different experimental approaches as described earlier.^33^ For detail, see Supplementary Methods.

## Results and Discussion

### Complete Genome Sequence of *P. stutzeri* ATCC 14405

To create an optimal basis for the downstream proteogenomic analysis, we sequenced and *de novo* assembled the first complete genome for a *P. stutzeri* ATCC 14405 strain (1 chromosome, no plasmids; Feb. 2019) and annotated it with NCBI’s PGAP (Figure S2) (see Methods).

Overall, the *P. stutzeri* genome (4639 Mb, 61.3% G + C content) was predicted to contain 4321 genes, including 148 pseudogenes, 61 tRNA, 12 rRNA, 4 ncRNA genes, and 4096 protein coding sequences (CDS) (Table S1). The 148 pseudogenes represent roughly three times as many as are annotated in the genome of *P. aeruginosa* MPAO1 (48), the parental strain of the widely used transposon insertion mutant collection,^34^ whose genome is ∼35% bigger (6375 Mb).^21^ Furthermore, the *P. stutzeri* genome encodes 74 transposases, that is, almost four times as many as annotated for MPAO1 (19).

Fifty of the annotated transposases were accounted for by multiple gene copies encoding an identical protein sequence (ranging from 2 (ISPsp6 family transposase) to 10 gene copies (e.g., IS30 family transposase)). The pseudogenes comprised 34 transposases, resulting in 108 transposases overall (Table S1). Notably, all seven lipase gene copies were pseudogenes suggesting that they are not required for *P. stutzeri* ATCC14405 in its natural habitat. As *P. stutzeri* is metabolically very diverse and broadly distributed in natural environments, a large effective population size has been postulated. Due to the very low recombination rates, bacterial clones can accumulate neutral mutations, which could lead to niche-specific selection and explain the large genotypic variation.^7^

Compared to a Roche 454 short read-based assembly of strain ATCC 14405 (RefSeq acc. GCF_000237885.1; 130 contigs),^18^ our complete genome sequence of 4,639,098 bp contained an additional 113,301 bp (Figure S2). This affected 144 genes (nine rRNA and nine tRNA genes, respectively) and 126 CDS that were either missed completely or only covered partially in the fragmented Roche 454 assembly. Notably, these included two cytochrome-c oxidase (*ccoO*) isoforms (Pstu14405_09650, Pstu14405_09665) contained within a cytochrome oxidase gene cluster, which we had also specifically sequenced in our structural analysis of the oxidase complex.^8^ The observation that a fragmented Illumina assembly can miss very important genes compared to a complete long-read-based assembly has recently been reported for *P. aeruginosa* MPAO1.^21^ Among 52 CDS not covered entirely in the MPAO1 Illumina assembly, four of eight (50%) nonribosomal peptide synthetases (NRPS) and three type VI secretion system effectors were affected underlining the value of complete genome sequences.

### Proteome Coverage by Bottom-Up and Direct Sequencing Tandem MS/MS Workflows

We next set out to identify the proteins expressed by *P. stutzeri* under aerobic and oxygen-limited conditions in order to gain insights into the proteome changes that play a role in denitrification, adaptation to low oxygen levels and thus potentially also for pathogenicity. To maximize our chances to identify novel small proteins not yet covered in the genome annotation, we applied both a standard bottom-up (shotgun proteomics) approach and our direct-sequencing approach, which integrates information from full-length proteoforms (top-down) and peptide data from unspecifically generated fragments (Figures 1, S1, and 2). For the latter, using a size selection step (see Methods), we enriched for proteins below a molecular weight of 30 kDa. For these proteins, we assumed the direct-sequencing workflow to have some advantages over the bottom-up workflow, including the detection of small proteins that would not be identifiable following a tryptic digest or a digest with another protease: such proteins might either have too many cleavage sites or few to none, leading to peptides either too short (<7 aa) or too long (>40 aa) for detection by bottom-up proteomics, respectively.

**Figure 2:**
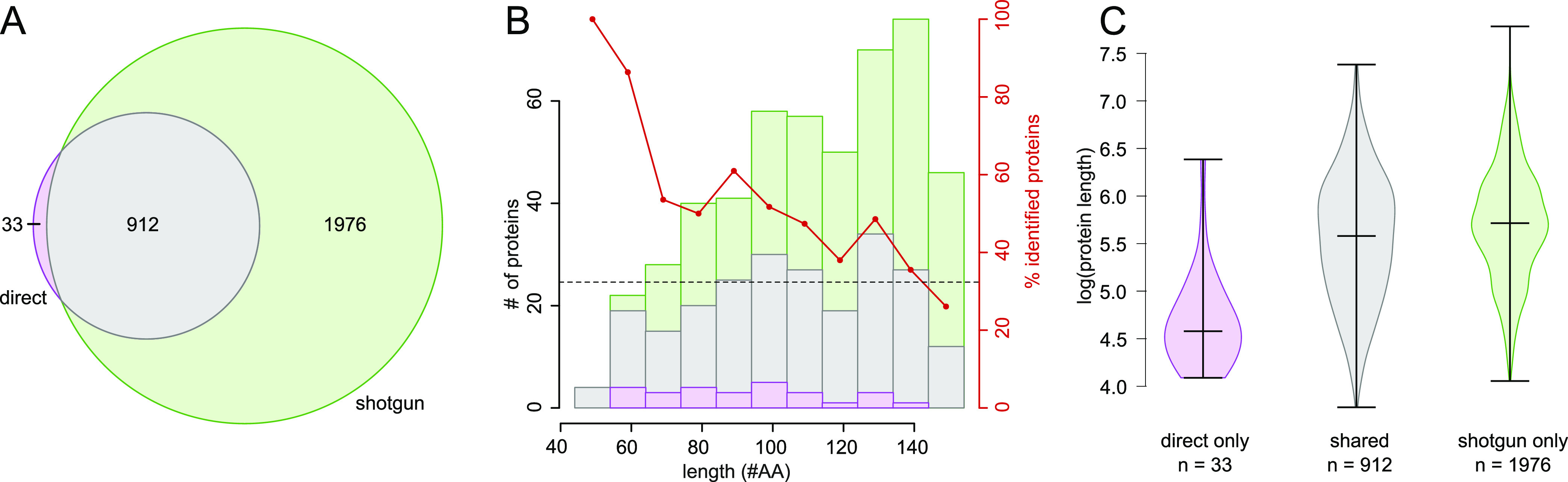
Proteins identified by shotgun proteomics and direct sequencing. Proteins identified either by direct sequencing or shotgun proteomics are shown in purple and green, those identified by both in grey, respectively. (A) Venn diagram for proteins identified with the bottom-up and direct-sequencing workflows. (B) Length histogram of RefSeq annotated proteins below 150 aa (the number of proteins is shown on the left *y*-axis). The percentage of proteins identified by direct sequencing compared to all identified proteins is shown as a red-dashed line overall and as red dots for each length bin (right *y*-axis). (C) Violin plot comparing the log protein length for the subsets 33 annotated proteins uniquely identified by direct sequencing, 912 proteins identified by both approaches, and 1976 proteins only identified by shotgun proteomics.

MS data from three independent biological replicates each grown under aerobic and oxygen-limited conditions were first searched against the NCBI RefSeq annotation-based protein DB (RefSeq 2019). A total of 2921 annotated proteins were identified in at least 2/3 replicates, that is, 71.3% of the predicted proteome (see Table S3, a master table that allows researchers to filter respective subsets and that contains numerous predictions and computations for all CDS). Overall, 2888 proteins were identified by shotgun proteomics and 945 by direct sequencing (about a third of those using bottom up), which largely overlapped (Figure 2A). Importantly, the direct-sequencing approach uniquely identified 33 RefSeq proteins that were predominantly small (Table S4). A bias analysis of physico–chemical parameters^33^ allowed us to establish that the direct-sequencing approach provided a benefit for the identification of short proteins below 140 aa. These accounted for 27 of the 33 proteins that were uniquely identified by direct sequencing (88.1%, Table S1, Figure 2B). Given that the direct-sequencing approach identified only about one-third of the proteins identified by shotgun proteomics (and overall), this underlines its substantial potential for small protein discovery. This becomes also evident when the length distribution of proteins uniquely identified by digest-free direct sequencing (33), those jointly identified by direct sequencing and bottom-up (912) and of the 1976 proteins uniquely identified by shotgun proteomics is compared (Figure 2C). Notably, for the abundantly expressed ribosomal proteins (52 overall, 75% shorter than 150 aa, Table S3), we observed that direct sequencing identified these proteins with a higher protein coverage and more peptides (Figure S3). Among the 2921 annotated proteins identified (Table S3), expression evidence was also observed for 28 of the 126 CDS that were missed in the fragmented Roche 454 assembly. This included proteins encoded by genes with an assigned gene name, such as both cytochrome-c oxidase CcoO isoforms (Pstu14405_09650, Pstu14405_09665) that differ in 6 out of their 203 aa, electron transport complex subunit RsxC (Pstu14405_15960), glycerol kinase GlpK (Pstu14405_08555) as well as several transposases, that would have been missed entirely.

### Differential Protein Expression in Aerobic and Oxygen-Limiting Growth

We then studied how *P. stutzeri* remodels its proteome under oxygen-limited conditions compared to normal aerobic growth. To this end, we made use of a custom setup that passaged either air or nitrogen gas into the bacterial cultures.^9^ We performed three independent experiments and compared protein abundances in both conditions using label-free quantitation (LFQ).

Among the 2888 proteins identified with shotgun proteomics, we quantified 2438 proteins, 809 of which were up- or down-regulated under either aerobic or oxygen-limiting conditions (PEAKSQ, 1% FDR; Figure S4). In addition, we identified 367 proteins exclusively in one condition in at least two of three samples (with one peptide) and in none of the technical or biological replicates in the other condition (Table S3).

**Figure 3:**
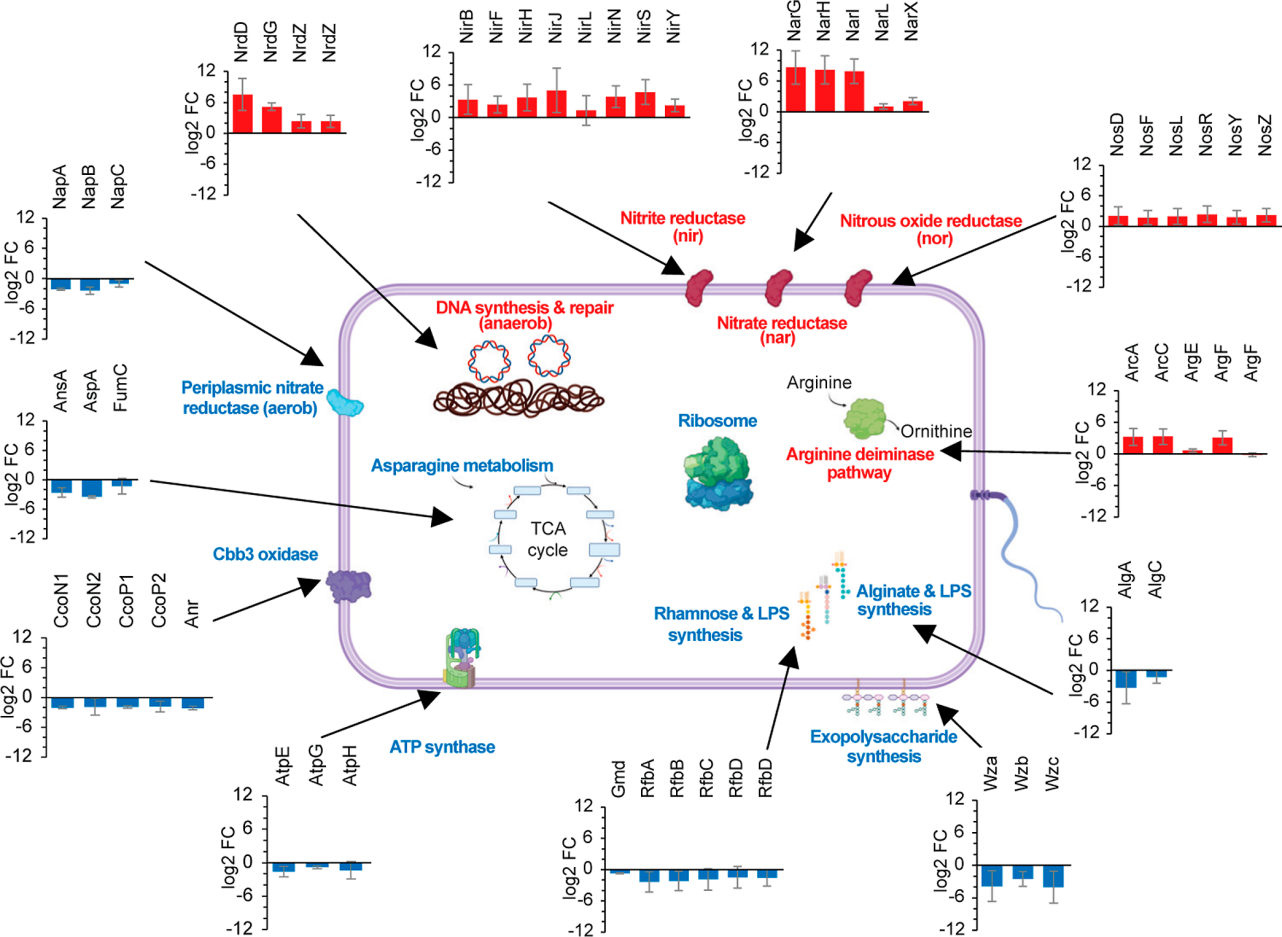
Differential protein expression under aerobic and oxygen-limited conditions. log2 fold changes of selected biologically relevant proteins or cellular processes/pathways are visualized in a scheme of a *P. stutzeri* cell (created with Biorender). Protein complex subunits that do not have enough unique peptides for quantification are omitted from this graph (e.g., CcoO isoforms, AtpA, B, C, and D).

We first evaluated our data by examining the differential abundance of proteins known to play essential roles under oxygen-limited conditions, where *Pseudomonas* changes its respiratory machinery from oxygen to nitrogen compounds as electron acceptors. The main denitrification pathway consists of the *nir*, *nar*, *nor,* and *nos* gene clusters, which encode the main enzymes involved in stepwise nitrogen reduction.^7^ Most of the annotated denitrification proteins displayed a significant up-regulation under oxygen-limiting conditions, such as NirJ [log2 fold change (FC): 5.01], NarG (log2 FC: 8.63), NorQ (log2 FC: 2.54), and NosZ (log2 FC: 2.19). As expected, the metabolic enzymes displayed a more pronounced effect than the auxiliary proteins and transcription factors (Figure 3). We also observed increased abundances for enzymes in the anaerobic arginine deiminase pathway (ArcA: log2 FC 3.2, ArgF: log2 FC 1.34; Figure 3), which is involved in fermentation of amino acids as an energy source. In addition, we found that multiple enzymes encoded by the *nrd* gene cluster involved in anaerobic DNA replication and repair were upregulated, which is in line with previous studies.^35,36^ Previous reports on *P. aeruginosa* also indicate a reduced Fe-uptake under hypoxic stress conditions.^10^*P. stutzeri* encodes 22 TonB receptors (compared to 34 TonB receptors in *P. aeruginosa*), and a similar trend was observed for some of these candidates. Overall, however, iron uptake does not seem to be affected under oxygen-limited conditions in *P. stutzeri*. However, we also added sufficient amounts of trace elements (including Fe(III), Cu(II), Mg(II) and Zn(II)) to our growth media to avoid any starvation during exponential growth. Similarly, proteins known to be essential for aerobic respiration were upregulated under aerobic conditions, such as respiratory dehydrogenases and reductases that feed substrates into the ubiquinol pool (indicated by negative log2 FCs, e.g., Ndh log2 FC -2.1; NqrB log2 FC: −1.43).

We also found that overall metabolic activity appeared to be higher: the protein synthesis machinery was upregulated in general, with a statistically significant increase in abundance for 48 of the 52 detected ribosomal subunits (Table S3). These observations are in line with a faster growth rate observed for *P. stutzeri* under aerobic conditions.^9^ In addition, we observed a pronounced increase in polysaccharide and lipo-polysaccharide synthesis pathways, specifically in the *rfb*, *wz,* and *alg* gene clusters, which play a role in cell wall biogenesis and thus directly reflect faster cellular growth.

Several essential enzymes for asparagine-dependent energy metabolism were strikingly up-regulated, such as the aspartate ammonia lyase AspA (log2 FC: −3.45) and the asparaginase AnsA (log2 FC: −2.58). Similarly, the periplasmic nitrate reductase NapA, a functional marker for aerobic denitrification,^37^ was upregulated under aerobic conditions (NapA log2 FC: −2.08). This metabolic adaptation is a direct result of and concurs with the asparagine-containing growth medium used in these experiments. Furthermore, we found that the ATP synthase cluster showed higher abundance under aerobic conditions, with four of eight detected subunits displaying a significant increase in abundance and all subunits showing a similar trend (e.g., AtpE log2 FC: −1.61; AtpH: log2 FC: −1.35; Figure 3). These findings are in good agreement with earlier studies on *Pseudomonas* respiration, including proteomics studies on *P. aeruginosa* isolates.^10,38^

We then investigated the terminal respiratory oxidases, a central element of the aerobic respiratory chain, in more detail. Previous studies suggested that the *cbb*_*3*_ cytochrome *c* oxidases in *P. aeruginosa* could play an essential role under micro-aerobic conditions, due to their high affinity to oxygen.^39^ Surprisingly, we found that the two *cbb*_*3*_*-type* cytochrome *c* oxidase isoforms (CcoN, CcoP) displayed significantly higher abundance under aerobic conditions, with similar effects for both isoforms (Figure 3). These observations are in distinct contrast to previous studies in *P. aeruginosa*, where *cbb*_*3*_ cytochrome *c* oxidase was found to be upregulated under microaerobic conditions and hypoxic stress.^10,39^ We speculate that our oxygen-limited conditions led to a full switch to the denitrification cascade and respective downregulation of any oxygen-utilizing oxidases, which also is in agreement with the observed up-regulation of the Anr protein, the regulator which controls *cbb*_*3*_-type oxidase expression (log2 FC: −2.13).^38^

Taken together, our results provide a comprehensive overview of the proteomic adaptations of *P. stutzeri* under oxygen-limited conditions.

### Analysis of Known Small Proteins

Overall, we detected 160 annotated small proteins <100 aa, that is, roughly 50% of the 323 proteins that are annotated in RefSeq2019 within that size range. These small proteins include comparatively well-characterized enzymes such as the sulfur-carrier protein ThiS (66 aa) and the Fe-S assembly protein IscX (66 aa), but also a large number of hypothetical and domain-of-unknown-function (DUF)-containing proteins (see below). Some of the annotated small proteins were differentially abundant, including several proteins associated with aerobic respiration that displayed higher abundance under this condition: for example, the small subunit CcoQ of the *cbb*_*3*_ cytochrome *c* oxidase (61 aa), showed distinctly higher abundance under aerobic conditions (Table S3), which matched the observed up-regulation of the other larger *cbb*_*3*_ cytochrome *c* oxidase subunits (Figure 3). Conversely, a 51 aa protein (QOZ94689.1; Pstu14405_04655) annotated by RefSeq as oxygen-sensitive reductase was only expressed under oxygen-limited conditions: 22 peptides and 113 PSMs were detected in our direct-sequencing experiments, 3 peptides and 28 PSMs in our bottom-up experiments. These data strongly suggest an increased abundance and physiological role under oxygen-limited conditions.

Interestingly, several small proteins for which no previous indication of a function in respiration or associated processes had been made displayed pronounced differential abundances under aerobic and oxygen-limited conditions. These included several hypothetical proteins with conserved “domains of unknown function” (DUF), such as a DUF3509-containing protein of 92 aa (QOZ94270.1; Pstu14405_02310). The protein was only detected under aerobic conditions and exclusively by our direct-sequencing approach; the 66 PSMs represent an extremely high value for a small protein. While they are widely conserved in prokaryotes (4503 entries for DUF3509 containing proteins in TrEMBL), no previous functional information is available for DUF3509 motifs, making this the first report of such a protein with a phenotype.

Our direct-sequencing approach also enabled the detection of proteoforms and PTMs that are inaccessible to trypsin-digestion-based analyses. For example, we detected a small protein of 59 aa which is heavily modified post-translationally (QOZ95262.1; Pstu14405_07830). Due to several N-terminal tryptic cleavage sites, no MS-detectable peptides (typically within the range from 7 to 40 aa upon a tryptic digest)^40^ from this region were found in bottom-up experiments. In our digest-free approach, though, in addition to the full-length protein, we also observed multiple N-terminal peptides with either alanine A1 or lysine K3 methylation (Figure S5). Notably, we only detected singly methylated peptides and no peptides in which both residues were modified at the same time. This small protein, which is highly conserved in *Pseudomonas*, is thought to play a role in RNA-binding in other *Pseudomonas* strains (homologue protein: 30S ribosomal protein S11, A0A078LSS1 (Uniprot)), for which this methylation could play a critical role. The protein was slightly upregulated under aerobic conditions (log2 FC: −0.72) but we were not able to determine significant changes in its modification state between growth conditions.

### Identification and Analysis of Novel Small Proteins

We next sought to identify novel small proteins and their potential function(s). For this, we generated an iPtgxDB that captures the entire protein coding potential of a prokaryotic genome^23^ and searched our bottom-up and direct-sequencing proteomics data.

Overall, we uniquely detected 16 additional, so far missed novel small proteins below 100 aa (Table S5, Figure S6). Thirteen additional unannotated ORFs coding for proteins ranging in size from 121 to over 800 aa were also detected, that would have been missed in a conventional UniProt database search (Table S6). A PeptideClassifier analysis (see Methods) ensured that all novel proteins (Tables S5 and S6) were unambiguously identified by one to several peptides (class 1a peptides).^17^ This information is critical, as—compared to the RefSeq2019 database (4096 proteins)—the large iPtgxDB contains over 128,000 potential proteins. While this captures almost the entire protein coding potential of the genome, the percentage of shared peptides increases substantially. In two cases, our peptide evidence code indicated a novel protein that will require further clarification: for a 67 aa in silico predicted protein exclusively identified by shotgun (Table S5), one class 2a peptide unambiguously identified a novel annotation cluster and supported expression of a novel proteoform, but we could not identify the precise N-terminus (Figure S7). For a novel 154 aa protein, we observed several class 3a peptides, which indicate an unambiguous protein identification that however can be encoded by several distinct genetic loci, in this case nine gene copies predicted to encode a transposase (Table S6, Figure S8).

**Figure 4:**
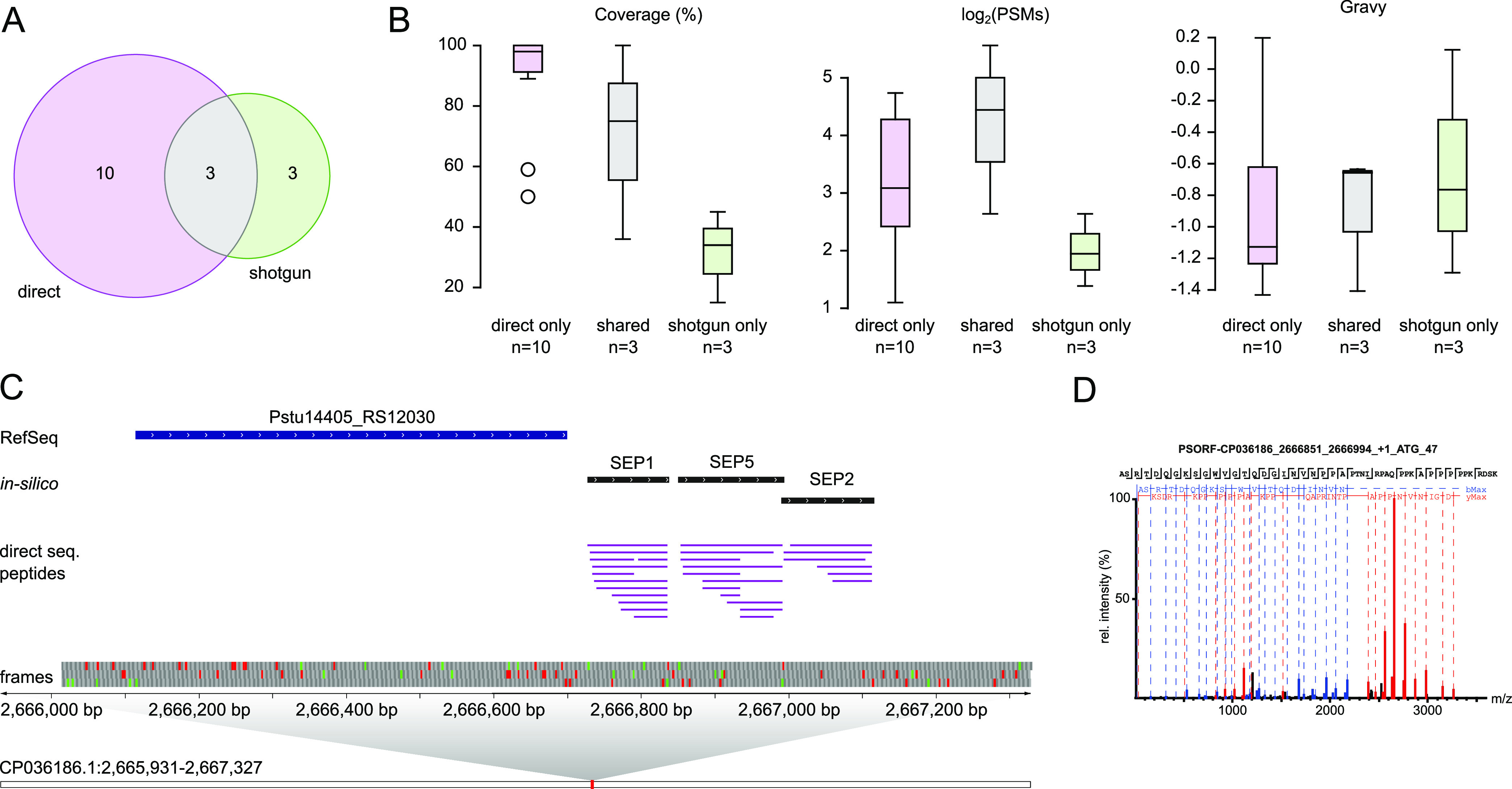
Analysis of 16 novel small proteins identified by direct-sequencing and/or shotgun proteomics. (A) Venn diagram summarizing the origin of MS-identified novel small proteins. (B) Protein coverage, PSM count, and gravy values represent parameters where substantial differences were observed. (C) Putative novel operon predicted by OperonMapper containing a RefSeq annotated gene (Pstu14405_RS212030; FUF6338-containing protein) and three novel short ORF-encoded proteins (SEPs) < 50 aa. Multiple peptides for each novel SEP were detected by direct sequencing while no peptides were found for the annotated protein in the two conditions. Pstu14405_RS12030 and SEP2 are encoded on frame +3, SEP1, and SEP5 on frame +1. D. An exemplary high-quality spectrum for “SEP5” indicated in panel C.

Among the 16 novel proteins, 3 were uniquely identified by shotgun proteomics, 3 were identified by both approaches, and 10 were uniquely identified by direct sequencing (Figure 4A). Notably, the sequence coverage for proteins identified by direct sequencing was substantially higher, reaching 100% for a few cases (Figure 4B), and they were identified with a substantially higher number of PSMs than the shotgun proteomics identifications (Figure 4B). Moreover, the novel proteins were enriched for proteins with very low grand average hydrophobicity (gravy) values (Figure 4B). As we had not analyzed membrane fractions, proteins with higher gravy values which are typical for membrane proteins,^33^ were under-represented among all identified proteins and the novels. The novel proteins also included two proteins that were annotated as pseudogenes in the latest RefSeq2019 release, but which became bona fide CDS in RefSeq2022. Notably, among the 16 novel small proteins, 9 were contained in the latest RefSeq2022 annotation release, lending further support to the quality of our data. All nine small proteins are annotated as hypothetical proteins (Table S5), that is, we here provide first evidence that they are truly expressed small proteins. This observation suggests that the genome annotation improved considerably in terms of coverage of small proteins. Yet, the seven novel small proteins with good experimental support that were missed in RefSeq2022 document that there is still substantial room to further improve the important genome annotation step.

Finally, we assessed the predicted function and conservation of the 16 novel sORFs, which included seven small proteins below 50 aa and nine below 100 aa (Table S5). Compared to the 13 longer novel proteins (Table S6) where robust functional predictions were uncovered, a putative function is only predicted for a few and with lower confidence. Eight novel small proteins, that were exclusively detected by direct sequencing, displayed a more than twofold higher proline content (Figure S9, Table S5), which may indicate disordered segments or roles as anti-microbial proteins.^41,42^ Proline-rich segments are generally less accessible to tryptic cleavage, potentially leading to under-representation in conventional bottom-up proteomics data. Three of these proline-rich and novel small proteins are predicted to form an operon with Pstu14405_12030 (Figure 4C/D), which is widely conserved in bacteria and harbors a DUF6338 domain that is also found in biosynthetic gene clusters. For our digest-free experiments, no intensity-based quantification is available due to insufficient MS1 features in our acquisition strategy. To estimate higher or lower abundance, we thus used an approximation via spectral counting (Table S3). The three novel proteins were well expressed under both aerobic and oxygen-limited conditions (ranging from 44 to 114 PSMs, approximately twofold up aerobically). They were only found in *P. stutzeri* and are not conserved beyond. Clearly, further experiments are required to elucidate a potential function of the encoded novel small proteins. Interestingly though, they might represent an example for the evolution of new genes.^43^

## Conclusions

Our extensive, genomics-driven study identified and quantified ∼2800 proteins involved in anaerobic or normal respiration of *P. stutzeri* and illustrates the benefits of a combination of the well-established bottom-up (shotgun) proteomics approach and digest-free direct sequencing, particularly for the identification of novel small proteins. Several new small proteins, which have not been described previously, showed strong differential regulation between the conditions. All data, including the first complete genome, are released as a resource for the community. Digest-free direct-sequencing and top-down proteomics are developing into highly useful alternatives in the mass spectrometry toolbox and we foresee major benefits particularly for the identification of novel small proteins, a research area that is gaining a lot of momentum. The combined approach is generically applicable to other prokaryotes.

## Data Availability

The *P. stutzeri* ATCC 14405 genome sequence (Genbank CP036186.1; BioProject PRJNA522963, BioSample SAMN10961665) and read data are available (Illumina: SRR8587050 and SRR8587051; PacBio: SRR8587049). The mass spectrometry data are available from PRIDE (dataset identifier PXD037914 and 10.6019/PXD037914). The iPtgxDB for *P. stutzeri* ATCC 14405 is available from https://iptgxdb.expasy.org, both as a searchable protein database (FASTA format) and a GFF file.

## Supporting Information Available

Details of selected experimental steps; integrated experimental workflow; genome map of *P. Stutzeri*; coverage for ribosomal proteins; bottom-up IDs and quantification; example spectra and coverage for N-terminal methylations; spectra implying novel small proteins; novel CDS with unclear N-terminus; with multiple copies; proline content of proteins; overview of genome features; data resources consolidated in the iPtgxDB; 33 known small proteins uniquely identified by direct sequencing (PDF)
Extensive master table (XLSX)
Table S5: Short novel small proteins (XLSX)
Table S6: Long novel small proteins for reference (XLSX)
