# Supplementary material for: Detection of Known and Novel Small Proteins in Pseudomonas stutzeri Using a Combination of Bottom-Up and Digest-Free Proteomics and Proteogenomics
Source: Anal Chem. 2023 Aug 3;95(32):11892–900. doi: 10.1021/acs.analchem.3c00676 (PMC10433244; doi:10.1021/acs.analchem.3c00676)
Supplement: Supplementary file 1 — ac3c00676_si_001.pdf [file ac3c00676_si_001.pdf]

Supporting Information

Detection of known and novel small proteins in *Pseudomonas stutzeri* using a combination of bottom-up and digest-free proteomics and proteogenomics

Jakob Meier-Credo[1], Benjamin Heiniger[2], Christian Schori[2], Fiona Rupprecht[3], Hartmut Michel[4], Christian H. Ahrens[2*], Julian D. Langer[1,3*]

[1] Proteomics, Max Planck Institute of Biophysics, 60438 Frankfurt am Main, Germany

[2] Molecular Ecology, Agroscope & SIB Swiss Institute of Bioinformatics, 8046 Zürich, Switzerland

[3] Proteomics, Max Planck Institute for Brain Research, 60438 Frankfurt am Main, Germany

[4] Department of Molecular Membrane Biology, Max Planck Institute of Biophysics, 60438 Frankfurt am Main, Germany

* Correspondence: christian.ahrens@agroscope.admin.ch, julian.langer@biophys.mpg.de

Table of Contents

Supplementary Methods 2

Cell growth 2

Cell lysis, protein solubilization and tryptic digestion 2

Small protein extraction 3

Details for the de novo genome assembly and comparison 3

Mass spectrometry 4

Analysis of proteomic datasets 5

Additional bioinformatic data analyses and visualization 6

Table S1. Selected genome features of *P. stutzeri* ATCC 14405 and comparison 7

Table S2. Annotation clusters of the *P. stutzeri* ATCC 14405 iPtgxDB 8

Table S3. Master table 9

Table S4. Overview of 33 RefSeq proteins only identified by direct sequencing 10

Table S5. Overview over 16 novel small proteins smaller than 100aa 12

Table S6. Overview over 13 novel small proteins larger than 100aa 13

Figure S1. Integrated analytical workflow for an improved detection of known and novel small proteins 14

Figure S2: Genome map of P. stutzeri ATCC 14405 15

Figure S3. Coverage and number of peptide identification for ribosomal proteins 16

Figure S4. Differential protein expression under aerobic and oxygen-limiting conditions 17

Figure S5. Peptide coverage and example PSMs for methylations on QOZ95262.1 18

Figure S6. Example PSMs and peptide coverages for novel SEPs by direct sequencing and bottom-up 19

Figure S7 A novel SEP with several possible proteoforms 35

Figure S8. A novel SEP encoded by multiple identical genes 36

Figure S9. Proline content of novel small proteins 37

References 38

Supplementary Methods

Cell growth

P. stutzeri cells were grown as previously described by Xie et al.{Xie, 2014 #11} Briefly, 50 µl cells from a glycerol stock were plated on a lysogeny broth (LB) agar plate containing 100 µg/ml ampicillin and incubated at 32°C overnight. Subsequently, 50 ml LB medium in a 100 ml Erlenmeyer flask (50 µg/ml ampicillin) were inoculated and incubated overnight at 220 rpm and 37°C. The preculture (1 ml) was further sub-cultured into 100 ml asparagine medium (supplemented with 11.8 mM KNO_3 for anaerobic conditions) in a 250 ml lockable bottle. This subculture was grown for 24 h at 150 rpm and 32°C. The main culture was grown in a 2 l fermenter (Ochs) under continuous injection of either purified air or nitrogen at 150 rpm and 32°C. Before inoculation of the main-culture, 1 l asparagine medium was heated up to 32°C and purged with the respective gases until inoculation. For anaerobic growth 1.2 g KNO_3 were added to the culture at optical densities at 600 nm (OD600 nm) of 0.1, 0.3 and 0.7. The cells were harvested through centrifugation (5000g, 4°C, 10 min) at an OD_{600} nm of 1.6, flash-frozen in liquid nitrogen and stored at -80°C.

Cell lysis, protein solubilization and tryptic digestion

Cell pellets were dissolved at 200 mg/ml in MS-grade water, supplemented with Benzonase (Sigma) and protease inhibitors (Roche). Subsequent homogenization was carried out using a rod sonifier (Branson) for 3x1 min (30% power, 50% duty cycle) on ice.
Cell lysates (50 µl) were then mixed in a 1:1 (v/v) ratio with solubilization buffer (10% SDS (Sodium dodecyl sulfate), 100 mM TEAB (triethylammonium bicarbonate) pH 8.5) to a final volume of 100 µl, placed in the ultrasonic bath for 1 h and cleared by centrifugation (3000 g, 4 min). The supernatants were collected and reduced by addition of 4 µl TCEP (Tris(2-carboxyethyl)phosphine) for 30 min at 37°C, followed by alkylation with 8 µl IAA (Iodoacetamide) for 30 min in the dark. Tryptic digestion was performed on S-Trap mini columns (Protifi). Therefore, samples were acidified with 12 µl phosphoric acid (12%), diluted sevenfold in S-Trap binding buffer (100 mM TEAB in 90% methanol, 10% water) and loaded onto the columns. SDS was removed by three washing cycles with 400 µl S-Trap binding buffer prior to the digestion. 2 µg trypsin (Serva) were added to 125 µl digestion buffer (50mM TEAB, pH 8.5) and the column incubated overnight at room temperature. Resulting peptides were eluted stepwise in 80 µl 50 mM TEAB, 0.2% formic acid, 50% acetonitrile (ACN). The pooled fractions were diluted 1:1 with 0.1% trifluoroacetic acid (TFA) and desalted with C18-SPE cartridges (Biotage). After equilibration with 2 ml acetonitrile (ACN), 1 ml 50% ACN / 1% acetic acid and 2 ml 0.1% TFA the samples were loaded onto the cartridge,

washed with 2 ml 0,1% TFA and eluted with 500 µl 80% ACN / 0.1% TFA. The eluted fractions were dried using an Eppendorf concentrator (Eppendorf) and stored at -20°C.

Small protein extraction

Cell lysates (100 µl) were extracted with 400 µl UA20 buffer (20% ACN, 6 M Urea) for 10 min in an ultrasonic bath. Extracts were loaded onto Microcon 30 molecular weight cutoff membrane centrifugal filter units (Merck, Germany), spun for 20 min at 18000 g and washed with additional 400 µl UA20 buffer for 20 min at 18000 g. The flow-throughs were collected and diluted with 1 ml 0.1% TFA. The protein extracts were desalted and further purified with C18-SPE cartridges (Biotage). After equilibration with 2 ml ACN, 1 ml 50% ACN / 1% acetic acid and 2 ml 0.1% TFA the samples were loaded onto the cartridge, washed with 2 ml 0.1% TFA and eluted with 1 ml 80% ACN / 0.1%TFA. The eluted fractions were dried using an Eppendorf concentrator (Eppendorf) and stored at -20°C.

Details for the *de novo* genome assembly and comparison

Length filtered PacBio subreads (> 1 kbp) were extracted and *de novo* assembled with Flye (v2.3.6)[2] using an estimated genome size of 4.6 Mbp. The assembly was polished with Quiver (v2.3.0.140893; PacBio' s SMRT Portal) until single variant level was reached (2 iterations). To correct any potentially remaining assembly errors, data from 2 × 300 bp paired-end Illumina reads were mapped to the assembly using BWA MEM (v0.7.17)[3] and FreeBayes (v1.0.0; with parameters minimum alternate fraction: 0.5, minimum alternate count: 5 until single variant level was reached (2 iterations).[4] Circularity and completeness of the *de novo* assembly was verified by re-mapping the circular chromosome using Graphmap (v0.5.2).[5] Structural variations were called using Sniffles (v1.0.7)[6], quality parameters were calculated by QualiMap (v2.2.1)[7] and the entire assembly was manually inspected using the Integrated Genome Viewer.[8] The Illumina reads were assembled with Plasmid SPAdes (v3.13.0)[9] to check for additional smaller plasmids, which could have been missed due to the Blue Pippin size selection step. The start of the single bacterial chromosome was set 200 bp upstream of the *dnaA* gene. Several genome characteristics including length, %GC, coverage, number of annotated genes, pseudogenes, RNAs and CDS were compiled in Table S1 and compared to that of a fragmented short read-based assembly of strain ATCC14405 and to *P. aeruginosa* MPAO1. The assembly of *P. stutzeri* ATCC 14405 based on Roche 454 GS-FLX data (RefSeq accession GCF_000237885.1) was compared by aligning the 130 contigs to the single chromosome in our *de novo* assembly with BWA MEM. The genomcov option of BEDTools v2.29.2[10] was used to find regions not covered by any short read contigs. Overlaps between uncovered regions larger than 5 bp and annotated features were determined using BEDTools' intersect option. Genes partially or completely missing from the Roche 454 based

assembly, as well as pseudogenes and GC skew, were visualized together with the mapped contigs with Circos v0.69-8.[11]

Mass spectrometry

LC-MS measurements were carried out on an Ultimate 3000 nanoRSLC (Thermo Fisher) system, equipped with an Acclaim Pepmap C18 trap column (2 cm * 75 μm, particle size: 3 μm; Thermo Fisher) and a C18 analytical column (50 cm * 75 μm, particle size: 1.7 μm; CoAnn Technologies) with an integrated liquid-junction and fused silica emitter coupled to an Orbitrap Fusion Lumos mass spectrometer (Thermo Fisher).

Proteolytic digests and protein extracts were dissolved in 40 μl sample buffer (95% water, 5% ACN, supplemented with 0.1% FA) and trapped on column for 6 min at flow rate of 6 μl/min with loading buffer (98% water, 2% ACN, supplemented with 0.05% TFA). The following separations were performed with a flow rate of 250 nl/min and gradient elutions of buffer A (water, supplemented with 0.1% FA) and buffer B (80% ACN, 20% water, supplemented with 0.1% FA).

Bottom-up proteomics samples were eluted with consecutive linear steps from 4-33% B in 150 min, 33-48% B in 30 min, 48-90% B in 1 min followed by constant flow at 90% B for 5 min. Peptides eluting from the column were analyzed in data-dependent mode with MS1 scans acquired from 350-1400 m/z in the orbitrap (max. IT: 50 ms, AGC: 50%, resolution: 60K) at a fixed cycle time of 1 s. Precursors were automatically selected based on highest intensity and passing filter criteria: charge state (2-5), minimum intensity ($\geq 2.5*10^4$) and matching peptide isotope pattern. Selected precursors were isolated in the quadrupole (window: 1.4 m/z) and subjected to HCD fragmentation (NCE: 30%). Fragment spectra were recorded in the orbitrap (max. IT: 54 ms, AGC: 100%, resolution: 30K). A dynamic exclusion list was used with a cycle time of 30 s and 10 ppm mass tolerance.

Direct sequencing samples were eluted with consecutive linear steps from 4-33% B in 75 min, 33-65% B in 15 min, 65-95% B in 1 min followed by constant flow at 95% B for 11 min. Peptides and proteins eluting from the column were analyzed by two methods in data-dependent mode employing splitted MS1 windows for precursor selection as well as a modified BoxCar method.[12]

For the splitted windows, MS1 scans were acquired in the orbitrap with mass ranges from 350-800 m/z and 800-2000 m/z (max. IT: 50 ms, AGC: 50%, resolution: 120K) with individual fixed cycle times of 2 s. Precursors were automatically selected based on highest intensities and passing filter criteria: charge state (2-15) and minimum intensity ($\geq 2.5*10^4$). Selected precursors were isolated in the quadrupole (windows: 1.4 m/z (low mass range), 1.6 (high mass range)) and subjected to HCD fragmentation (NCE: 30%). Fragment spectra were recorded in the orbitrap (max. IT: 54 ms, AGC: 80%, resolution: 30K). A dynamic exclusion list was used with a cycle time of 30 s and 10 ppm mass tolerance.

For the modified boxcar method, survey MS1 scans were recorded with a mass range of 350-2000 m/z (max. IT: 50 ms, AGC: 50%, resolution: 120K) that was further split into bins of 45 m/z and distributed into three multiplexed tSIM scan events (max. IT: 118 ms, AGC: 400%, resolution: 60K) with a fixed total cycle time of 4.5 s. Precursors were automatically selected from the tSIM scans based on highest intensities and passing filter criteria: charge state (2-15) and minimum intensity ($\geq 2.5*10^4$). To enhance the detection of highly charged and/or high mass compounds, which were missed by the automated picking, an additional scan was enforced upon a detected charge state >5 or mass >1500 Da. Selected precursors were then isolated in the quadrupole (window: 1.6 m/z) and subjected to HCD fragmentation (NCE: 35%). Fragment spectra were recorded in the orbitrap (max. IT: 54 ms, AGC: 100%, resolution: 30K). A dynamic exclusion list was used with a cycle time of 60 s and 10 ppm mass tolerance.

Analysis of proteomic datasets

Mass spectrometry data files were processed with PEAKS Studio (version 10.6). *De novo* sequencing and consecutive database searches against the RefSeq and iPtgxDB databases were performed with mass tolerances of 15 ppm (MS1) and 0.02 Da (MS2) respectively. For bottom-up files, carbamidomethylation (cysteine, fixed), oxidation (methionine, variable), acetylation (N-term, variable) and formylation (N-term, variable) were set as modifications and trypsin selected as digestion enzyme, allowing up to three missed cleavages. For direct sequencing files, oxidation (methionine, variable), acetylation (N-term, variable) and formylation (N-term, variable) were set as modifications using an unspecific search approach. Resulting database matches were filtered with a 0.1% peptide spectrum match (PSM) level FDR for bottom up and 0.3% for direct sequencing to achieve an estimated protein level FDR below 1%. To increase stringency, only proteins with at least one PSM in at least 2/3 samples per growth condition were retained in the dataset.

To test if our method could potentially have a benefit for PTM analyses, we performed an additional screening with 312 built in modifications using PEAKS PTM to check for abundant modifications among our identified small proteins. In a second PTM search we then extended the search space with N-terminal as well as lysine methylations.

Label free quantifications, including TIC normalization, retention time alignment and ID transfer, were performed with PEAKS Studio on the bottom-up samples in a feature-based approach, requiring at least one unique peptide for a protein in 2/3 samples for both conditions. Significant fold changes were determined using PEAKSQ with an adjusted Benjamini-Hochberg FDR of 1%. We further extracted proteins detected only in one condition manually by spectral counting. These proteins are listed separately, with specific PSM counts indicated for each candidate. Functional protein annotations were obtained through EGGNOG-mapper.[13]

Additional bioinformatic data analyses and visualization

Physico-chemical parameters were calculated using BioPython[14] (v1.76) and biases of proteins identified by bottom-up and direct sequencing were visualized with Matplotlib[15] (v3.3.0) to highlight the respective benefits of different experimental approaches as described earlier.[16] Proteins uniquely identified by bottom up and direct sequencing and proteins identified under aerobic or oxygen-limiting conditions were visualized using matplotlib-venn (v0.11.5, https://github.com/konstantint/matplotlib-venn).

Table S1. Selected genome features of *P. stutzeri* ATCC 14405 and comparison

The genome annotation of our complete assembly (as of February 2019 = ”RefSeq2019”) was used to compile the RefSeq and iPtgxDB search databases. Below, we compare several features of the complete genome to that of the Roche 454 assembly of *P. stutzeri* ATCC 14405. These features are also shown for *P. aeruginosa* MPAO1 that is mentioned in the main text (despite having a substantially larger genome, the number of pseudogenes is 3 fold lower, that of transposases roughly 5 fold).

	***P. stutzeri* ATCC 14405**	***P. stutzeri* ATCC 14405 (Roche 454)**	***P. aeruginosa* MPAO1**
Genbank accession #	CP036186.1	GCF_00237885.1	CP027857
No. chromosomes (plasmids)	1 (0)	130 contigs	1 (0)
Genome size (bp)	4,639,098	4,525,589	6,275,467
G+C content (%)	61.3 %	61.4%	66.5 %
Coverage PacBio	78x	n.a.	180x
Coverage Illumina MiSeq	84x	20x (Roche 454)	101x
No. of genes	4321	4181	5926
No. of protein-coding genes (CDS)	4096	4126*	5799
No. of rRNA operons (16S, 23S, 5S)	4,4,4	2,2,2	4,4,4
No. of tRNA genes	61	104*	63
No. of nc RNA genes	4	n.d.	4
No. of pseudogenes	148	n.d.	48
No. of transposases (corrected)	74 (+34 add. pseudogenes)	37 (+n.d. add. pseudogenes)	14 (+5 add. pseudogenes)

*The higher numbers for CDS and tRNAs are due to the fragmented Roche 454 assembly, reported in the form of 130 contigs. n.a. = not applicable; n.d. = not determined

Table S2. Annotation clusters of the *P. stutzeri* ATCC 14405 iPtgxDB

Overview of the step-wise hierarchical integration of different genome annotations for an identical genome sequence. We used RefSeq2019 as highest quality resource and then sequentially added Prodigal, Chemgenome and finally *in silico* predictions (for more detail, see /https://iptgxdb.expasy.org/creating_iptgxdbs/).

Row three of the table for example indicates that Prodigal predicted 4266 CDS, 149 of which were not contained in RefSeq2019, and thus the same number of new clusters is added. Moreover, for the existing annotation clusters, Prodigal predicted 179 shorter proteoforms compared to RefSeq, and 223 longer proteoforms (for both sets, the stop codon coordinates are identical). At the end of this first step of the sequential integration, we end up with 4798 sequences and IDs, before integrating the next annotation source.

Finally, we also created an iPtgxDB with 128,431 proteins (protein search database, FASTA format). We excluded 3118 potential N-terminal extensions smaller than 6 aa, 147 entries (annotation clusters where the anchor sequence was a pseudogene) and 106 entries that would create an indistinguishable internal start site. The 4247 RefSeq entries included 4096 CDS + 148 pseudogenes + 3 fCDS entries, i.e., programmed frameshifts.

**Genome annotat. resource /prediction**	**No. annotations**	**Clusters**	**No. new clusters**	**No. new reductions**	**No. new extensions**	**Total clusters**	**No. total ids**
**RefSeq** (REFSEQ)	4247	4247	4247	0	0	4247	4247
**Prodigal** (PROD)	4266	4266	149	179	223	4396	4798
**Chemgenome** (CHEMG)	7112	7112	3967	79	1779	8363	10,623
***In silico* ORFs** (ORF)	143,515	78,030	69,706	359	51,114	78,069	131,802

Table S3. Master table

This table provides an integrated overview of the genomic information of protein coding genes (NCBI locus tag, genomic coordinates, protein length, UniProt ID), details the different proteomics datasets (2921 overall annotated proteins identified by proteomics, 945 identified by direct sequencing, 2888 by shotgun proteomics, 33 known proteins uniquely identified by direct sequencing (**Figure 2**); 367 uniquely expressed proteins, 323 annotated proteins below 100aa, 160 identified proteins below 100aa, 10 top-down unique novel small proteins, 3 novel small protein identified by direct sequencing and shotgun proteomics, 3 shotgun only identified novel small proteins (**Figure 4**); 52 ribosomal proteins) as well as numerous bioinformatic predictions (physico-chemical parameters, predicted subcellular localization (PSORTb), transmembrane and signal peptide domains, operon prediction (OperonMapper), conservation in other species, functional prediction from eggNOG, Phyre2 and other prediction tools). Finally, we also provide the match with the latest annotation (RefSeq 2022), where several of the novel small proteins we identified have been annotated in the meantime. See separate Excel table.

Table S4. Overview of 33 RefSeq proteins only identified by direct sequencing

For the uniquely identified proteins, Genbank locus tag, protein length, predicted isoelectric point, PSMs from oxygen-limiting and aerobic conditions, predicted signal peptide, TM domain and Genbank annotation (plus gene name, where available) are shown. 27 proteins (81.8%) are 140 aa or shorter, i.e., within the length range where direct sequencing exhibited a benefit for small protein discovery (see Results).

Accession (LocusTag)	length (aa)	pI	# PSMs ox.-limiting / aerobic	SignalP	TMHMM	Description
Pstu14405_00055	79	4.90	41/80			Type II toxin-antitoxin system RelB/DinJ family antitoxin
Pstu14405_01210	168	4.83	0/5 (aerob exclusive)			Type VI secretion system contractile sheath small subunit (tssB)
Pstu14405_01515	69	6.14	7/25			TIGR02449 family protein
Pstu14405_02310	92	6.79	0/66 (aerob exclusive)			DUF3509 domain-containing protein
Pstu14405_02325	101	4.01	0/16			Hypothetical protein
Pstu14405_02330	60	4.12	2/9	Yes		Hypothetical protein
Pstu14405_03410	190	8.48	0/2 (aerob exclusive)	Yes	Yes	HupE/UreJ family protein
Pstu14405_03550	138	5.32	13/9		Yes	Polyribonucleotide nucleotidyltransferase
Pstu14405_04545	113	7.08	4/1	Yes	Yes	Cytochrome c
Pstu14405_05285	60	9.70	2/4		Yes	Hypothetical protein
Pstu14405_06435	63	8.27	8/1			Hypothetical protein
Pstu14405_06470	126	4.49	13/76			DNA polymerase III subunit chi
Pstu14405_06865	105	4.46	0/4 (aerob exclusive)			DUF2288 domain-containing protein
Pstu14405_06930	98	4.50	9/8			Aspartate-semialdehyde dehydrogenase
Pstu14405_07145	308	6.13	6/9		Yes	Alpha/beta hydrolase
Pstu14405_07615	104	8.09	1/3	Yes		Helix-hairpin-helix domain-containing protein
Pstu14405_07770	596	4.93	0 /8 (aerob exclusive)	Yes	Yes	Peptidase S8 and S53 subtilisin kexin sedolisin
Pstu14405_08885	87	6.44	9/13			Hypothetical protein
Pstu14405_12625	133	4.78	2/1	Yes	Yes	Hypothetical protein
Pstu14405_13525	105	5.80	2/15			ArsR family transcriptional regulator

Pstu14405_13530	96	5.79	13/40			Metal-sensing transcriptional repressor
Pstu14405_13890	116	4.69	23/11			Hpt domain-containing protein
Pstu14405_13965	170	4.13	2/3			Hypothetical protein
Pstu14405_15170	65	6.78	2/4			Hypothetical protein
Pstu14405_15290	174	4.96	8/16			Ankyrin repeat domain-containing protein
Pstu14405_15610	84	5.48	3/1			Glutamate synthase
Pstu14405_16125	77	7.03	2/3			Hypothetical protein
Pstu14405_16140	97	8.80	5/17			Hypothetical protein
Pstu14405_16535	86	4.03	1/19			DUF2007 domain-containing protein
Pstu14405_16810	64	4.40	0/3 (aerob exclusive)			DNA gyrase inhibitor (yacG)
Pstu14405_19690	130	4.41	9/2	Yes		DUF4168 domain-containing protein
Pstu14405_20185	77	4.37	1/5			Hypothetical protein
Pstu14405_21185	70	5.28	11/17			XRE family transcriptional regulator possibly oxidative stress related

Table S5. Overview over 16 novel small proteins smaller than 100aa

This table is available as a separate Excel file.

Table S6. Overview over 13 novel small proteins larger than 100aa

This table is available as a separate Excel file.

Figure S1. Integrated analytical workflow for an improved detection of known and novel small proteins

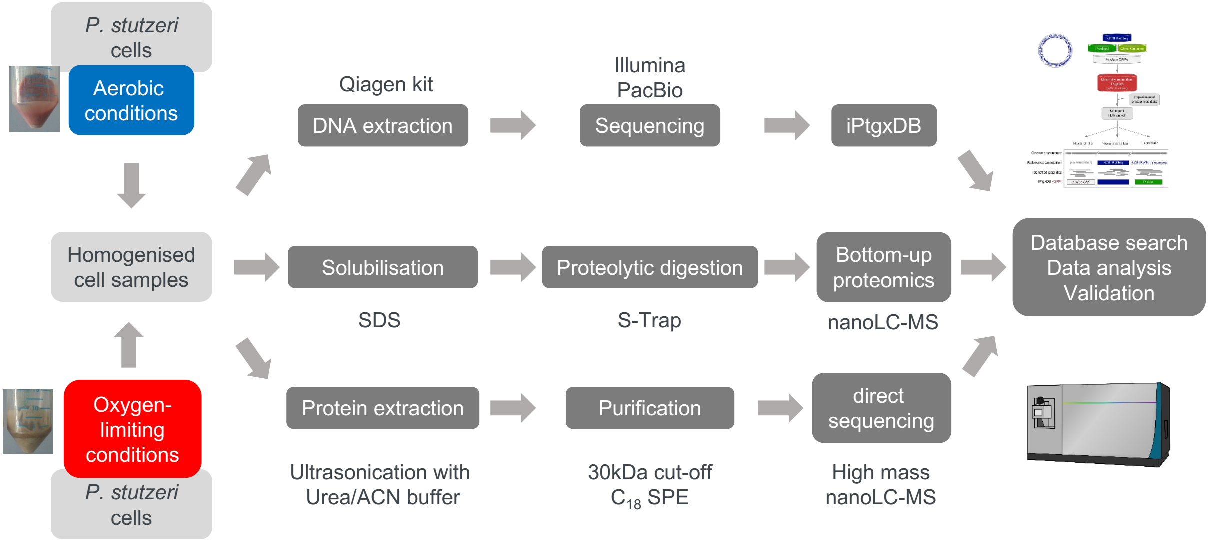

Figure S1. Our workflow integrates bottom-up and digest-free direct sequencing proteomics approaches in a proteogenomic framework. The *de novo* assembled complete genome of *P. stutzeri* ATCC 14405 provides an optimal basis for the identification of novel small proteins with an integrated proteogenomics search database (iPtgxDB) (upper row). Researchers can create such iPtgxDBs for their genome(s) of interest using a publicly available web server (https://iptgxdb.expasy.org/). From *P. stutzeri* cell extracts grown under aerobic (blue) or oxygen-limiting (red) conditions, both a shotgun proteomics (middle row) and a digest-free direct sequencing workflow (bottom row) were carried out, with the latter expected to have some advantages for the detection of small proteins. Data were then searched against a standard RefSeq database (not shown) and the large iPtgxDB that captures almost the entire protein coding potential of *P. stutzeri* ATCC14405.

Figure S2: Genome map of P. stutzeri ATCC 14405

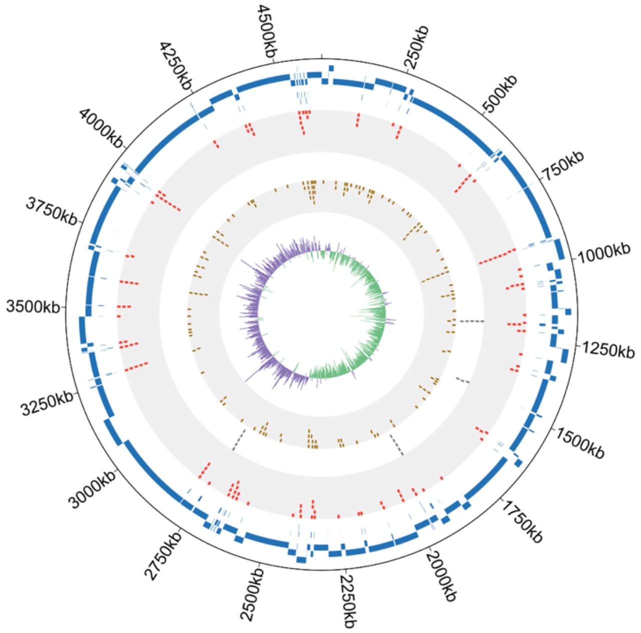

Figure S2. Comparison of the complete *de novo* assembly (outermost black circle) with a fragmented Roche 454 based assembly (blue contigs, second circle, and respective gaps, third circle, blue). Also shown are 144 genes not completely covered by the 130 contigs (126 CDS in red, fourth circle; tRNA and rRNA genes in gray, fifth circle), pseudogenes (sixth circle in brown) and the GC skew (assessed in 5 kb windows; innermost circle, purple for positive, green for negative).

Figure S3. Coverage and number of peptide identification for ribosomal proteins

A

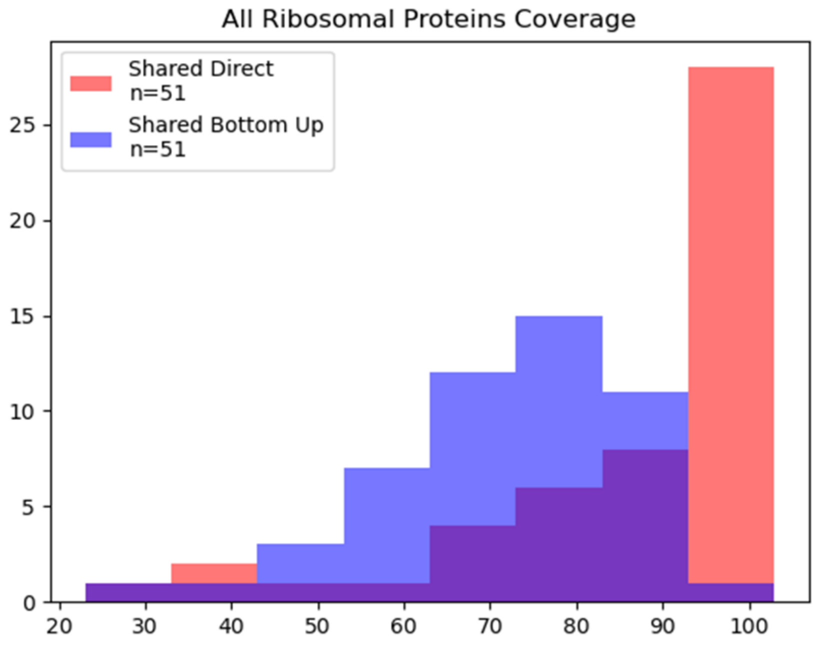

B

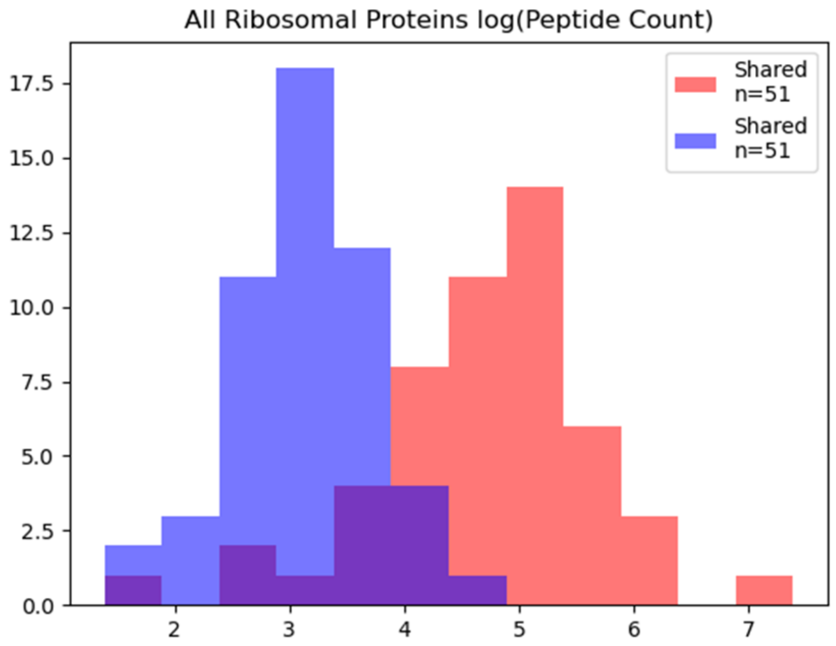

Figure S3. We compared A) the protein coverage achieved and B) the number of peptides identified with shotgun proteomics (blue) and direct sequencing (red) for this set of abundantly expressed proteins. Among 52 annotated ribosomal proteins (**Table S3**), 51 were identified by both methods, 1 protein was only identified by shotgun proteomics. Trends for greater protein coverage and a larger number of identified peptides per protein can be clearly observed for the direct sequencing data.

Figure S4. Differential protein expression under aerobic and oxygen-limiting conditions

A **B**

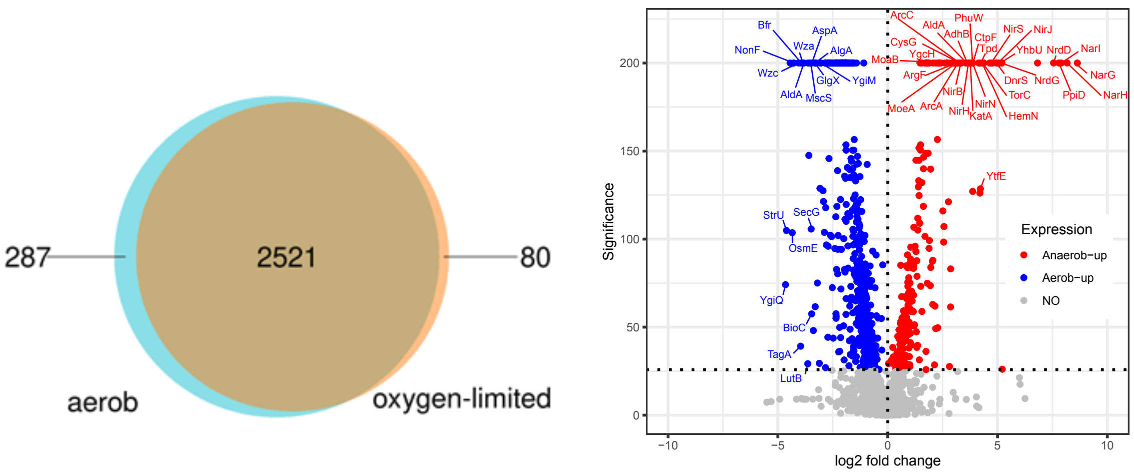

Figure S4. A. Venn diagram summarizing the proteins identified with the bottom up approach. B. Volcano plot of differentially expressed proteins (PeaksStudio); proteins significantly down-regulated under aerobic growth (blue) and upregulated under oxygen limitation (red) are shown. Proteins in the top part are highly significant and represented in an overflow bin. Significantly regulated proteins with a log2 fold change >3 are indicated with their predicted gene names.

Figure S5. Peptide coverage and example PSMs for methylations on QOZ95262.1

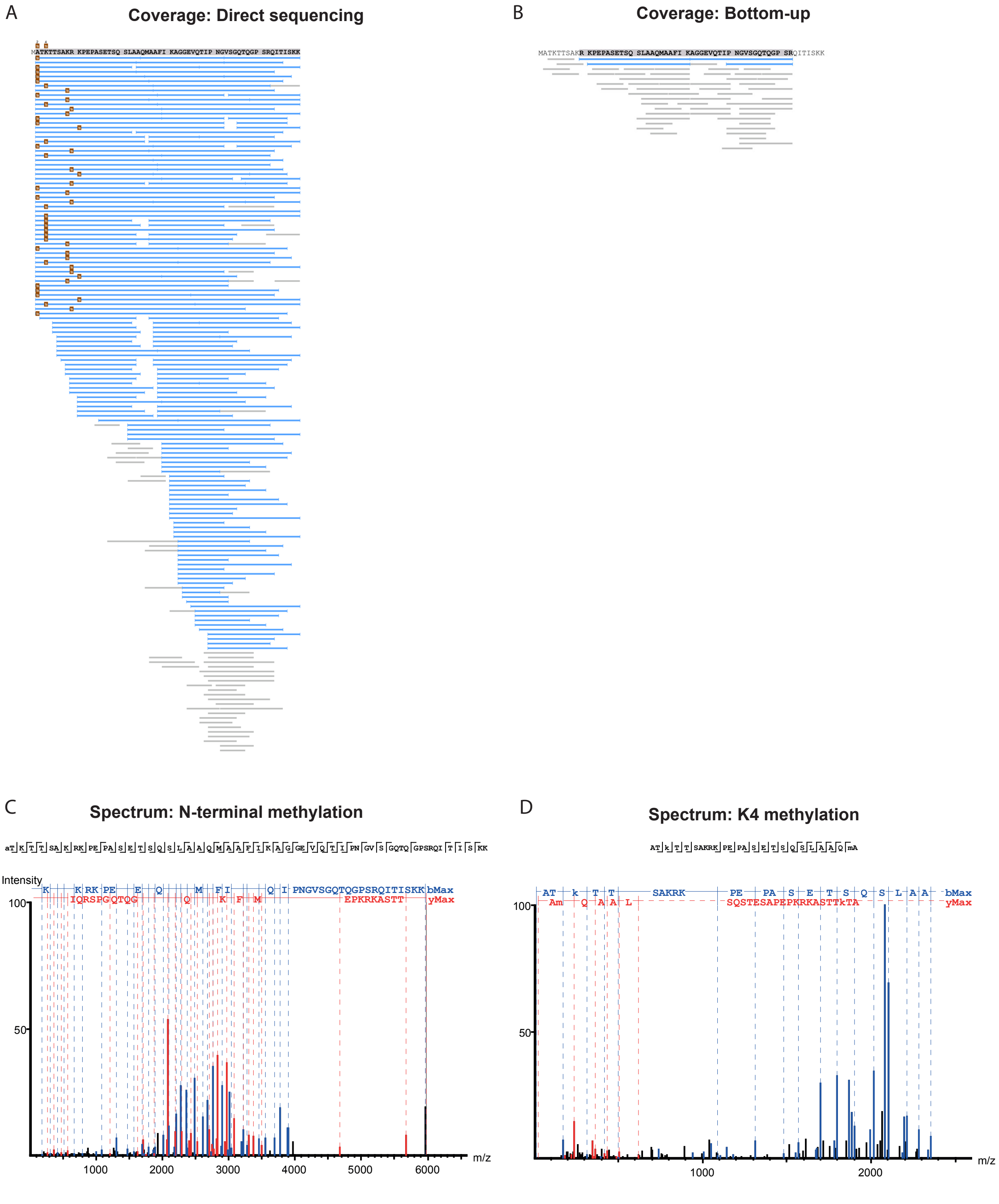

Figure S5. Peptide coverages obtained by direct sequencing (A) and bottom-up (B) methods for QOZ95262.1. Blue bars represent peptide matches, while grey bars show matching de novo tags. Detected methylation sites are marked with brown boxes. Example PSMs are shown for methylations on the N-terminus (C) and on K4 (D)

Figure S6. Example PSMs and peptide coverages for novel SEPs by direct sequencing and bottom-up

SEP1

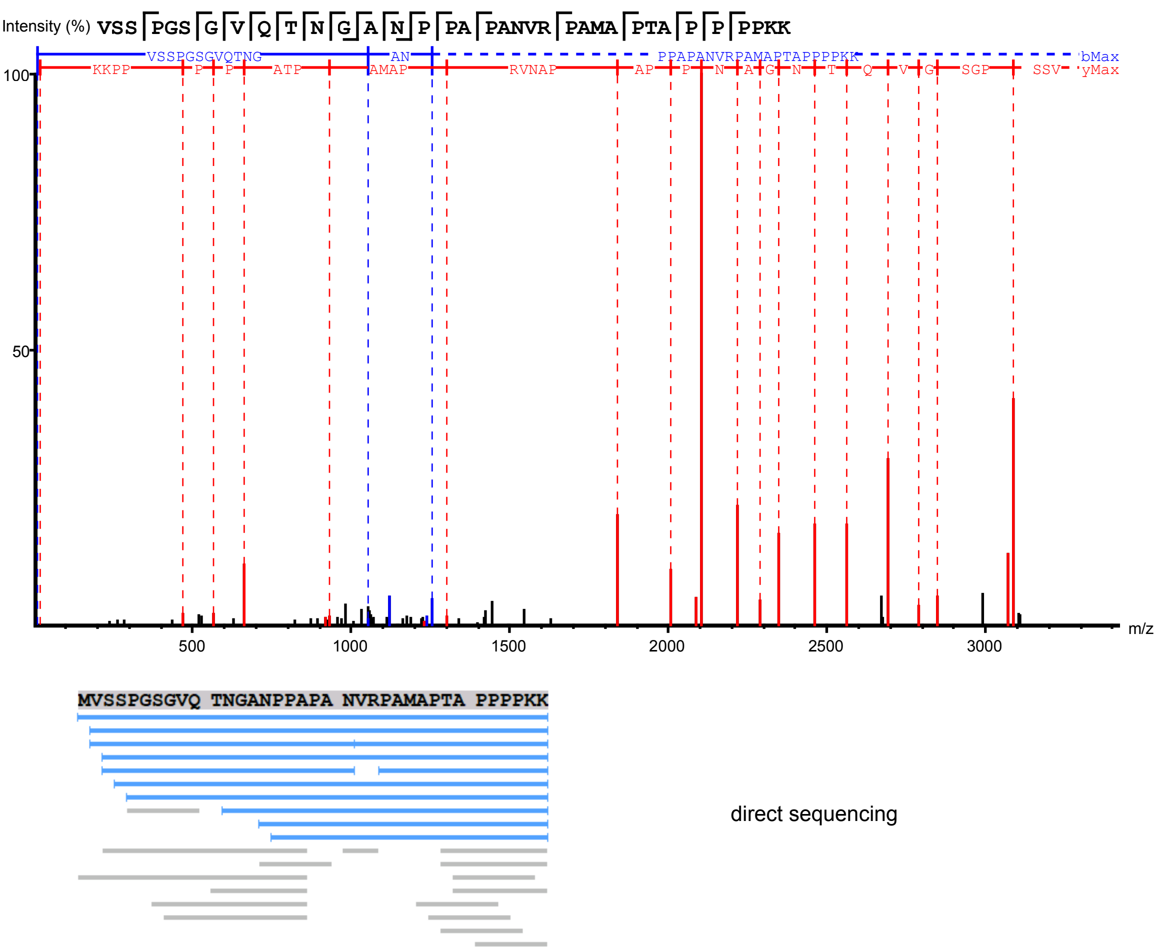

SEP2

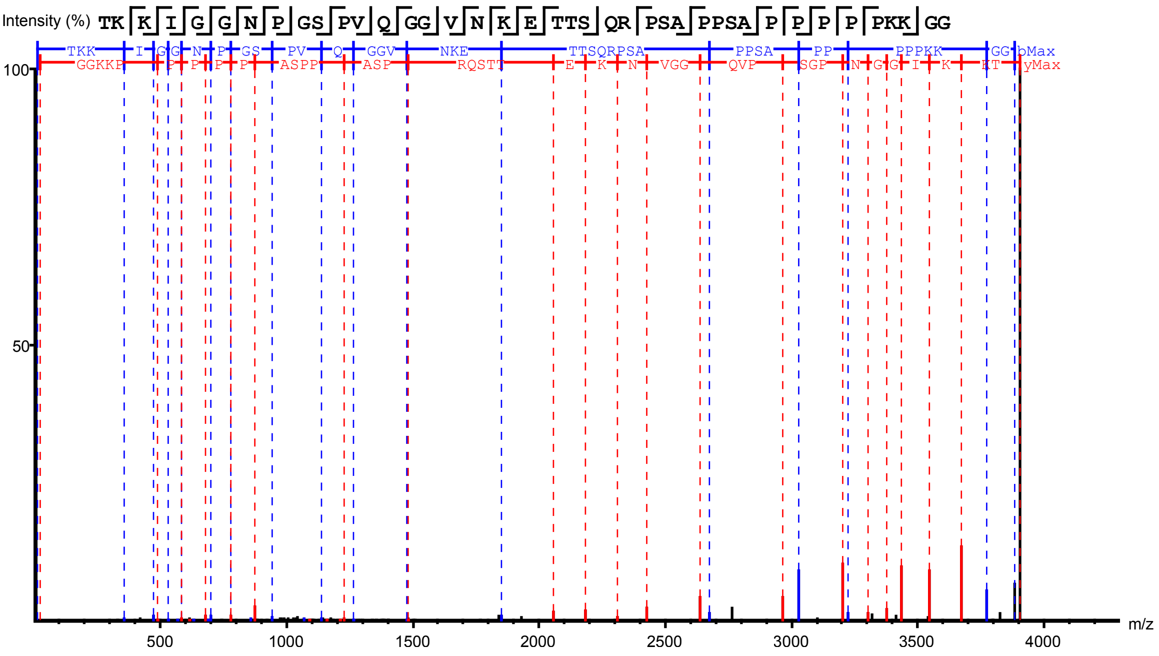

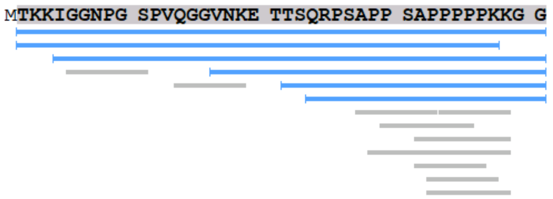

direct sequencing

SEP3

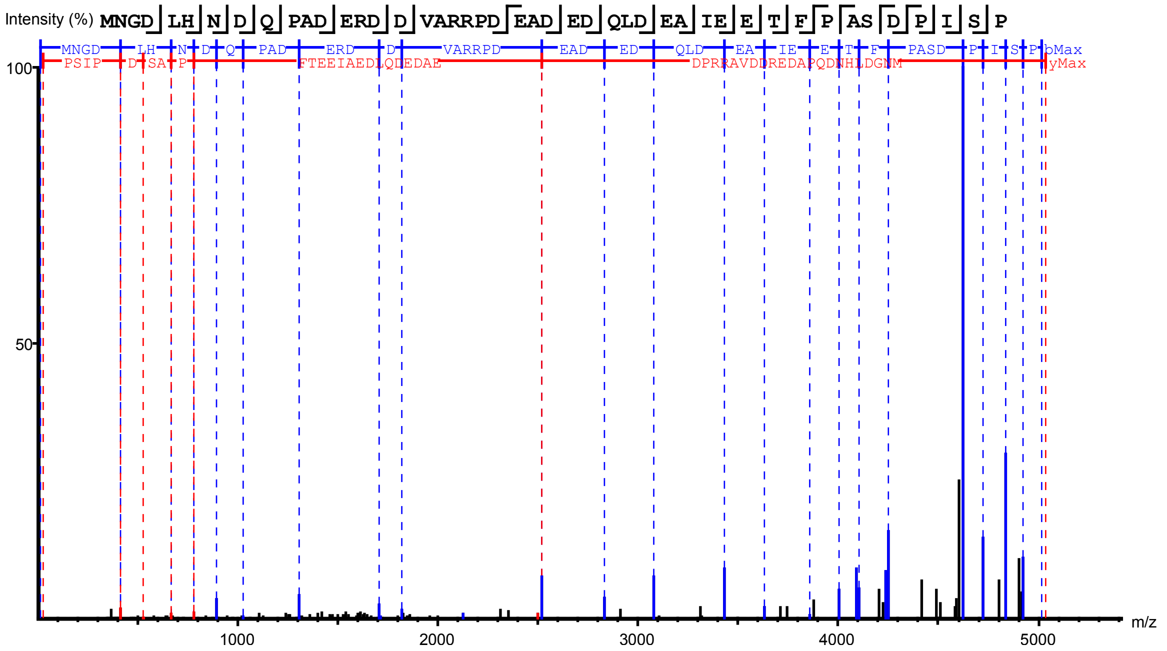

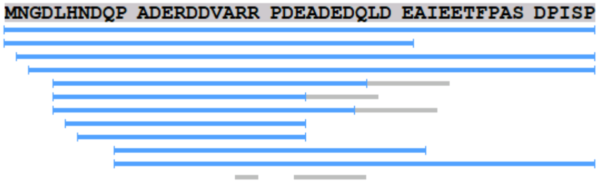

direct sequencing

SEP4

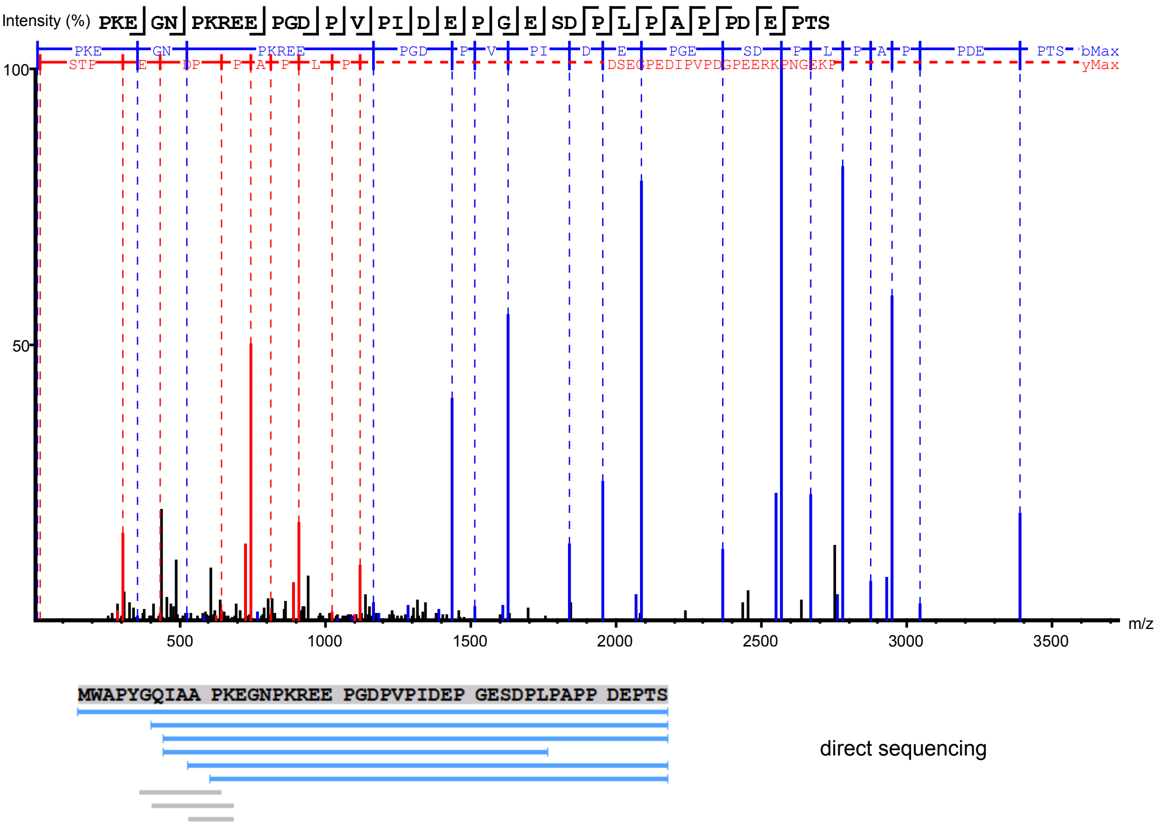
Intensity (%)
PKE GN PKREE PGD P V PI D E P G E SD P L P A P PD E PTS
bMax
yMax
100
50
500
1000
1500
2000
2500
3000
3500
m/z
MWAPYGQIAA PKEGNPKREE PGDPVPIDEP GESDPLPAPP DEPTS
direct sequencing

SEP5

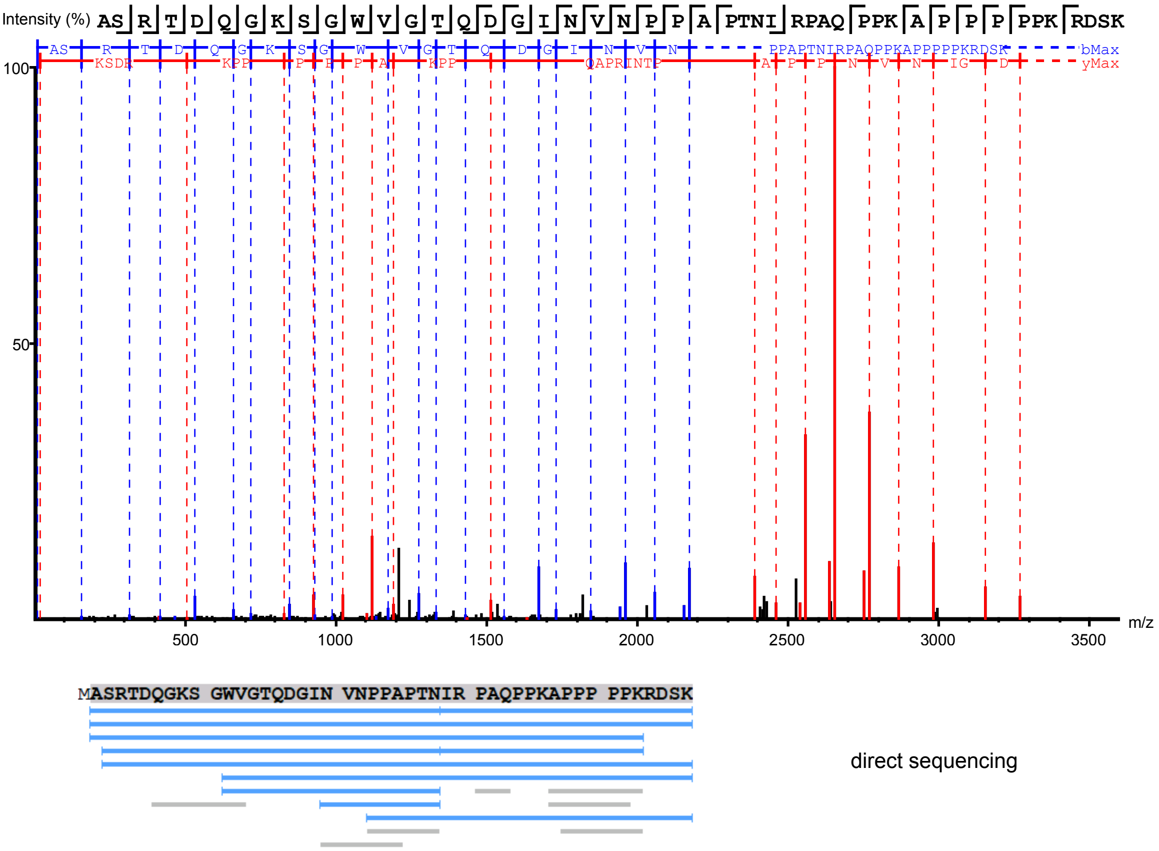

direct sequencing

SEP6

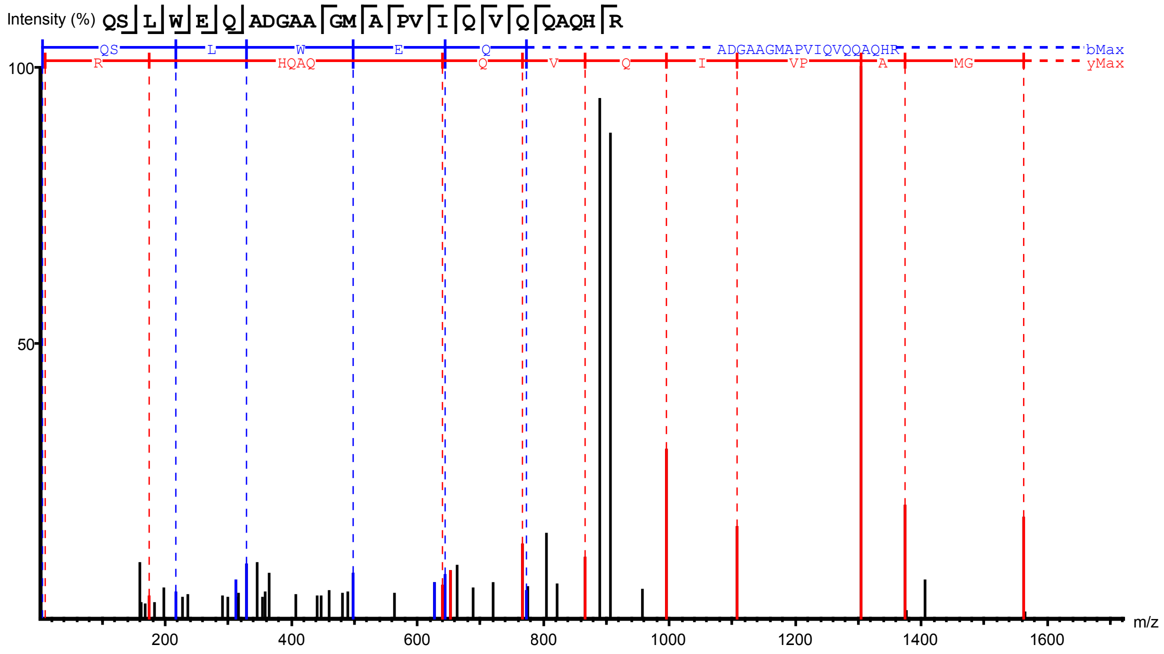

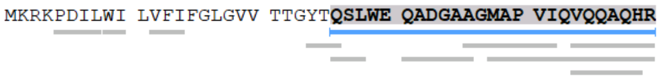

direct sequencing

SEP7

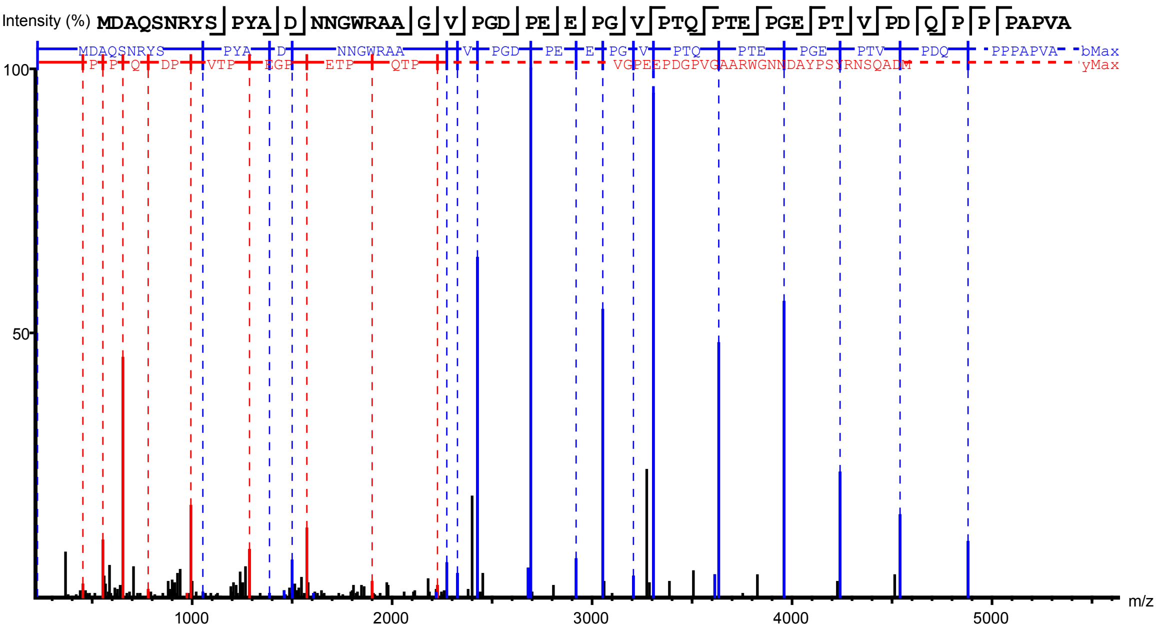

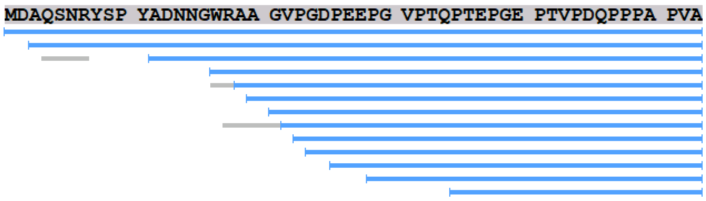

direct sequencing

SEP8

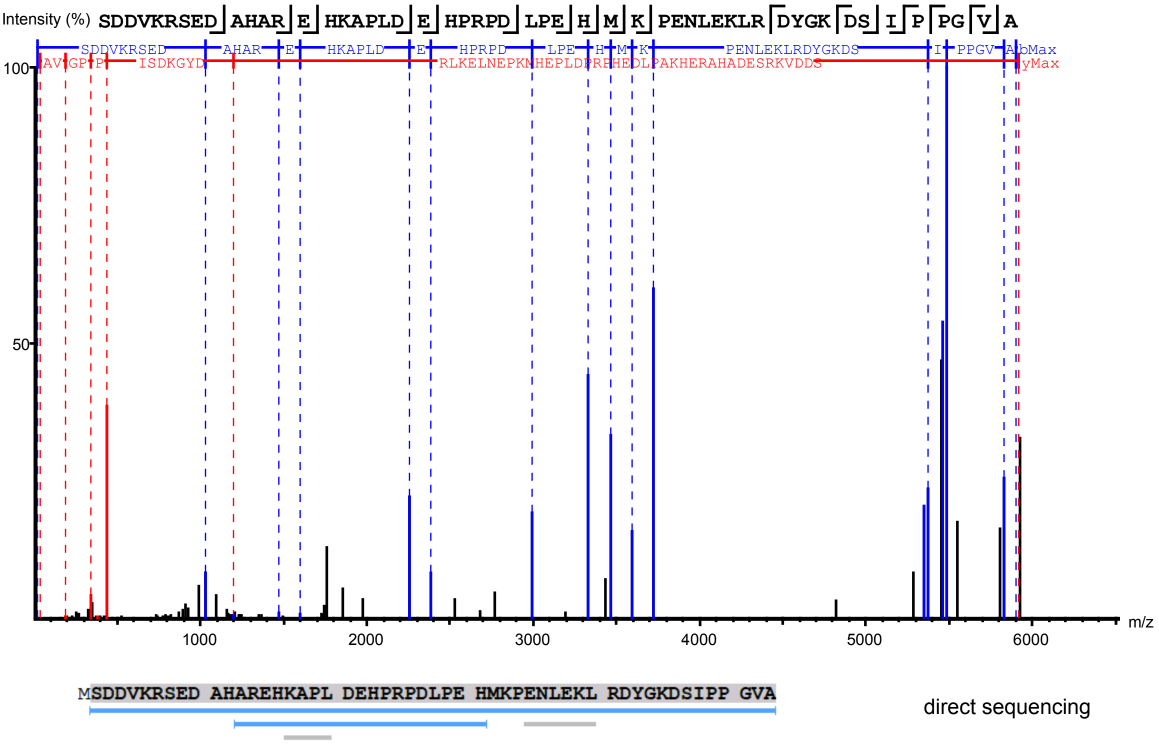
Intensity (%)
SDDVKRSED AHAR E HKAPLD E HPRPD LPE H M K PENLEKLR DYGK DS I P PG V A
bMax
yMax
100
50
1000
2000
3000
4000
5000
6000
m/z
MSDDVKRSED AHAREHKAPL DEHPRPDLPE HMKPENLEKL RDYGKDSIPP GVA
direct sequencing

SEP9

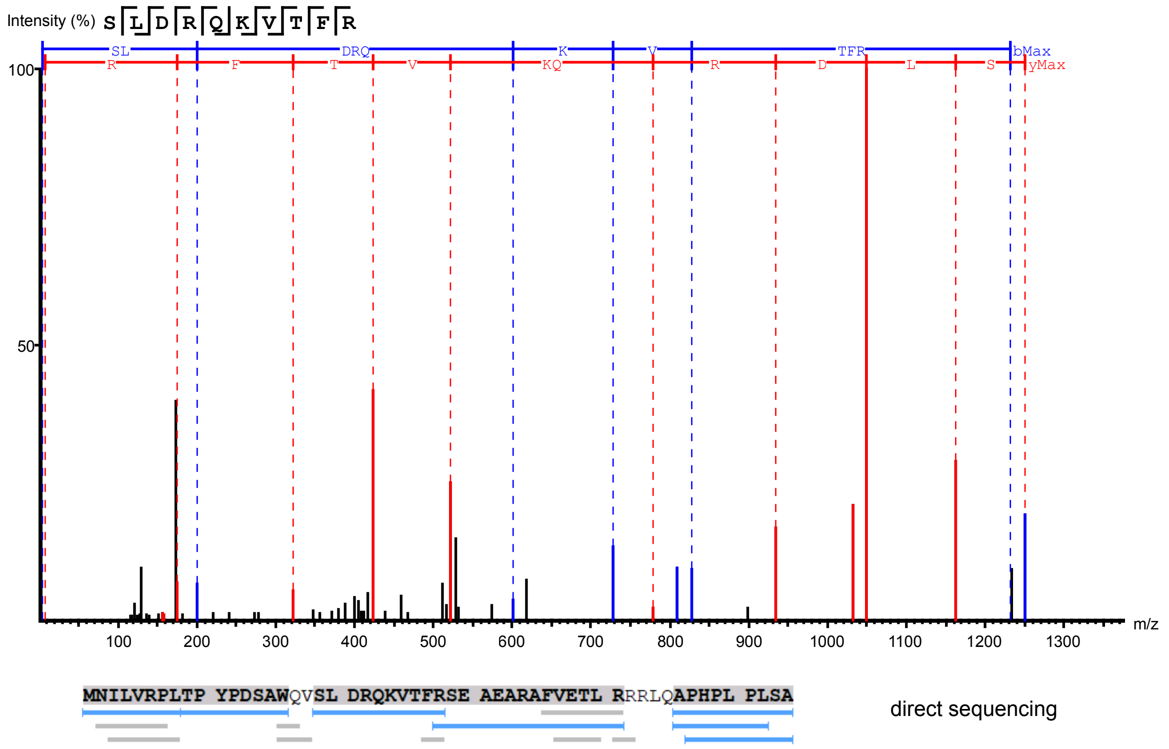

Intensity (%)
S L D R Q K V T F R
SL
DRQ
K
V
TFR
bMax
R
F
T
V
KQ
R
D
L
S
yMax
100
50
100
200
300
400
500
600
700
800
900
1000
1100
1200
1300
m/z
MNILVRPLTP YPDSAWQVSL DRQKVTFRSE AEARAFVETL RRRLQAPHPL PLSA
direct sequencing

SEP10

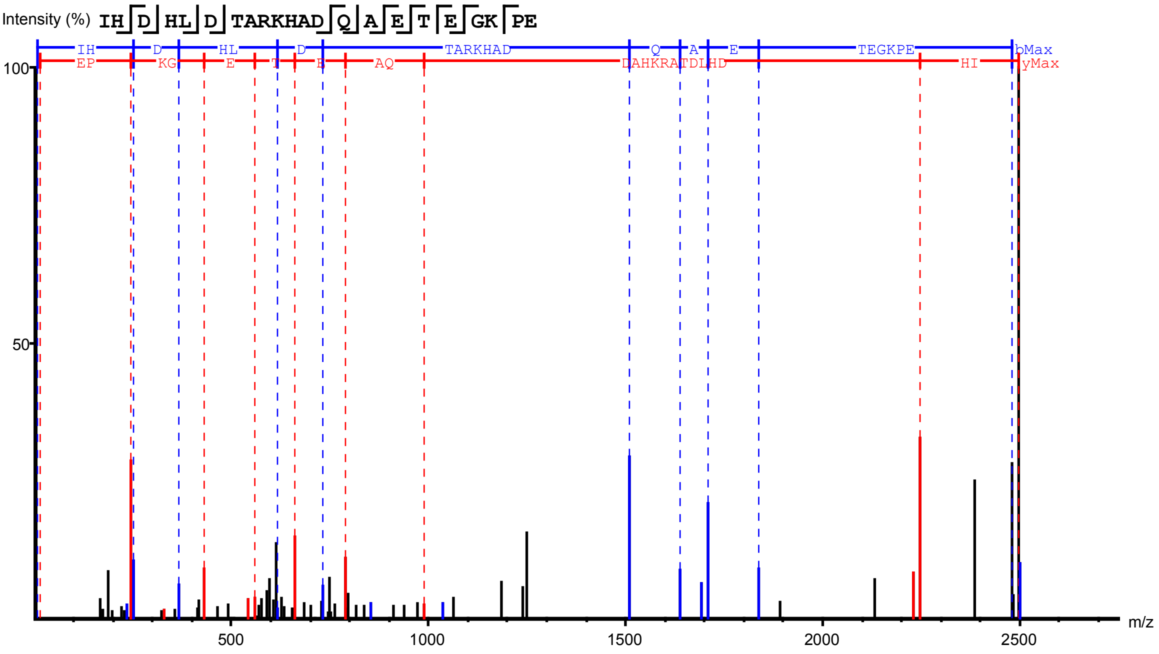

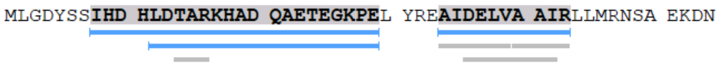

direct sequencing

SEP11

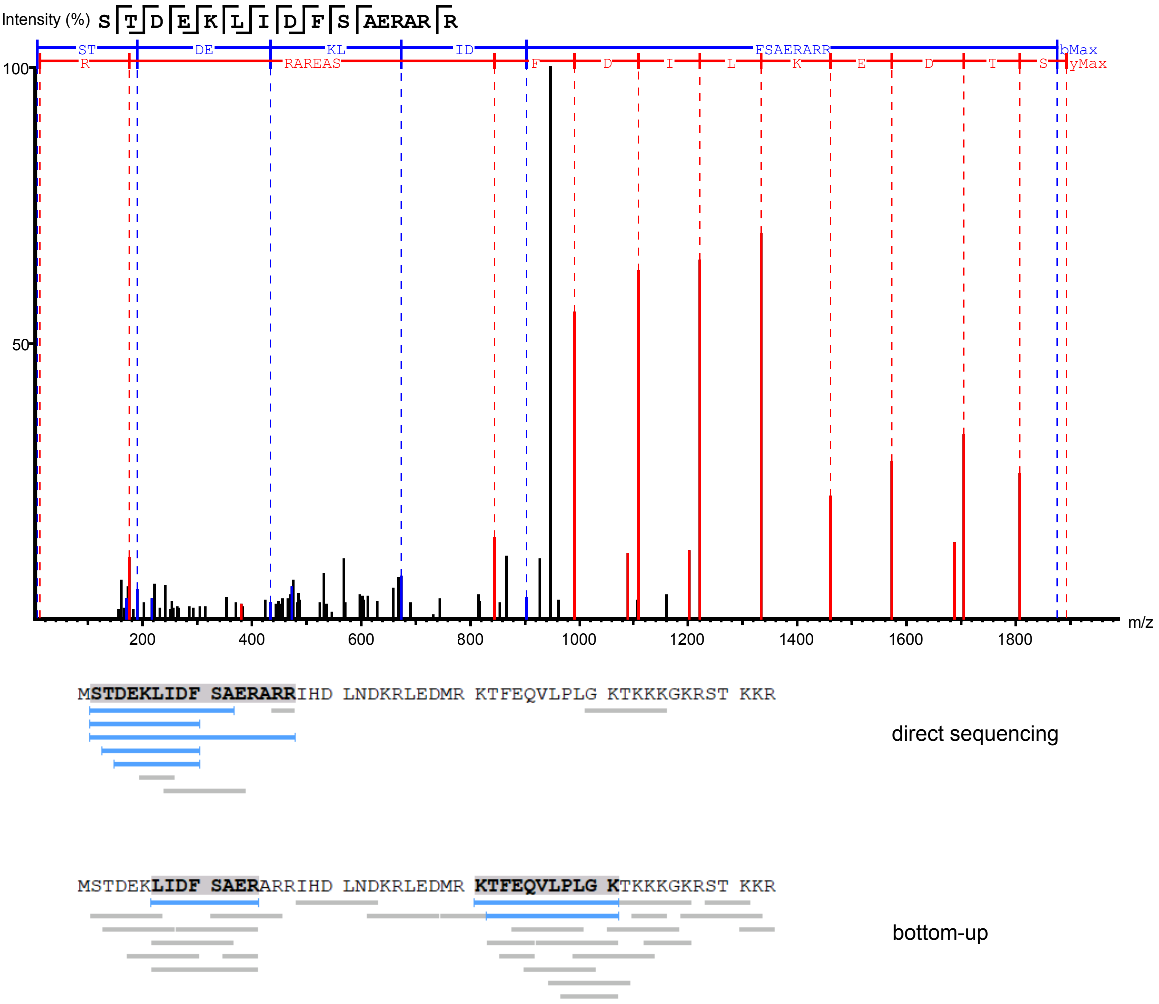
Intensity (%)
S T D E K L I D F S AERAR R
ST
DE
KL
ID
FSAERARR
bMax
R
RAREAS
F
D
I
L
K
E
D
T
S
yMax
100
50
200
400
600
800
1000
1200
1400
1600
1800
m/z
MSTDEKLIDF SAERARRIHD LNDKRLEDMR KTFEQVLPLG KTKKKGKRST KKR
direct sequencing
MSTDEKLIDF SAERARRIHD LNDKRLEDMR KTFEQVLPLG KTKKKGKRST KKR
bottom-up

SEP12

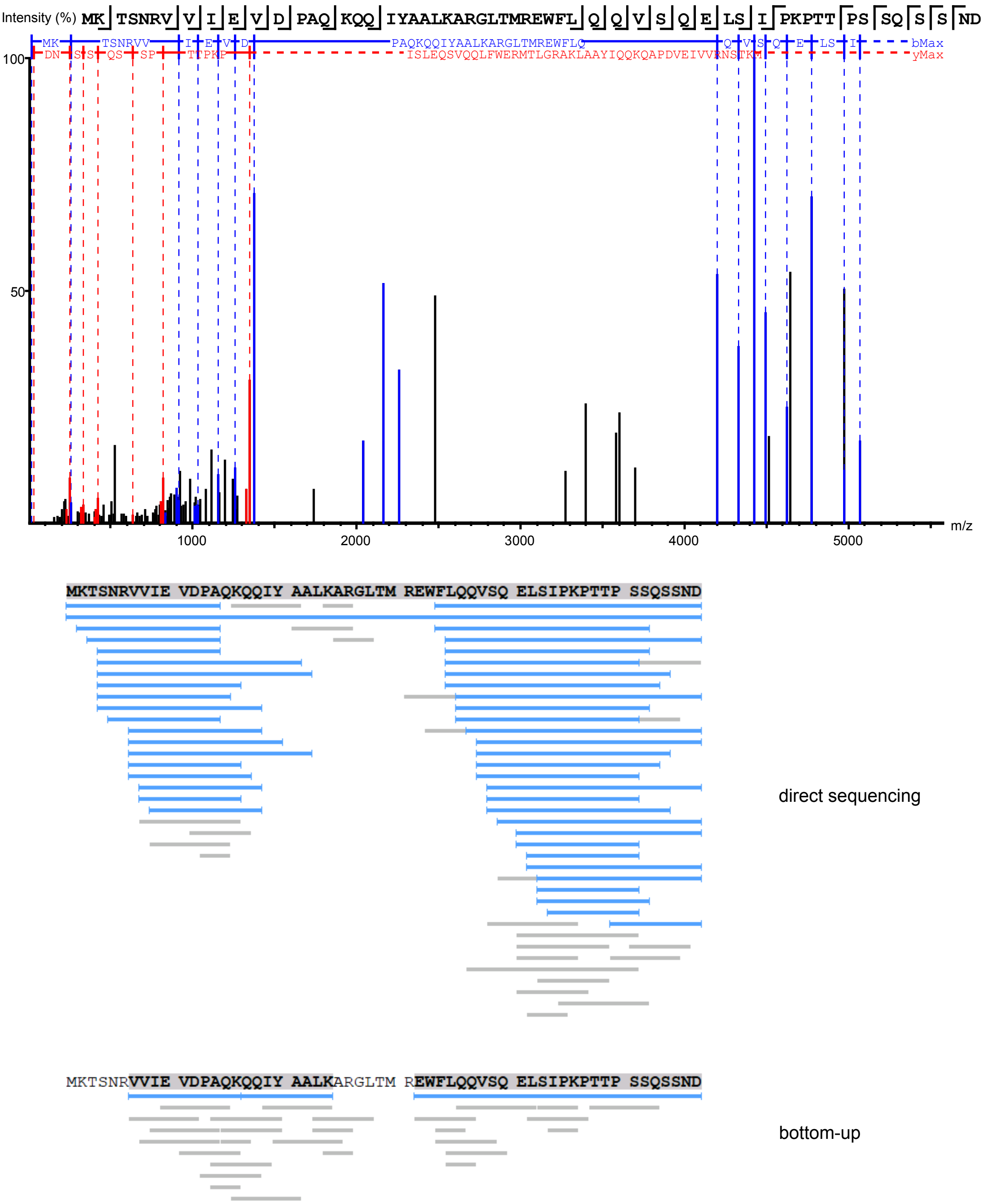

Intensity (%)
MK TSNRV V I E V D PAQ KQQ IYAALKARGLTMREWFL Q Q V S Q E LS I PKPTT PS SQ S S ND
bMax
yMax
100
50
1000
2000
3000
4000
5000
m/z
MKTSNRVVIE VDPAQKQQIY AALKARGLTM REWFLQQVSQ ELSIPKPTTP SSQSSND
direct sequencing
MKTSNRVVIE VDPAQKQQIY AALKARGLTM REWFLQQVSQ ELSIPKPTTP SSQSSND
bottom-up

SEP13

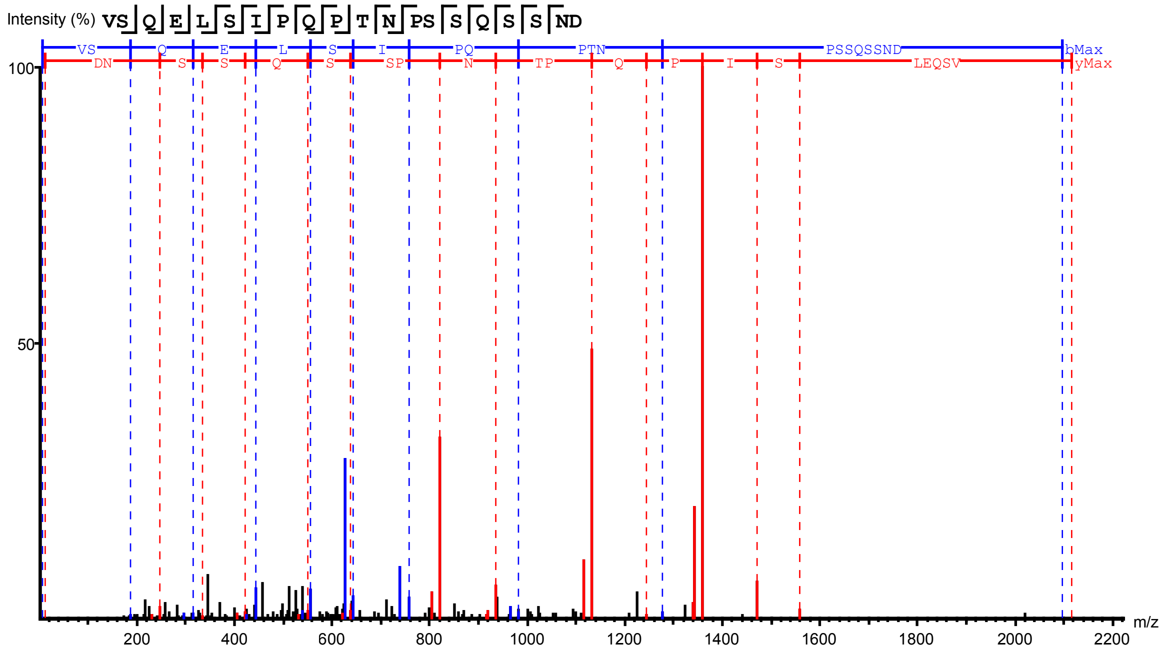
Intensity (%)
VS Q E L S I P Q P T N PS S Q S S ND
m/z
200 400 600 800 1000 1200 1400 1600 1800 2000 2200
100
50

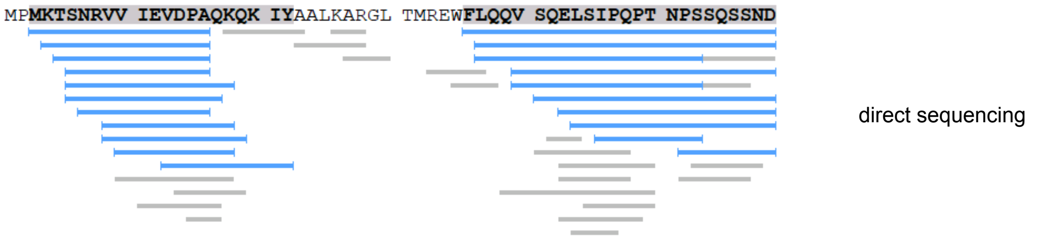
MPMKTSNRVV IEVDPAQKQK IYAALKARGL TMREWFLQQV SQELSIPQPT NPSSQSSND
direct sequencing

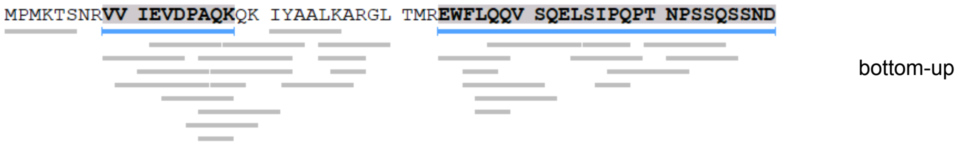
MPMKTSNRVV IEVDPAQKQK IYAALKARGL TMREWFLQQV SQELSIPQPT NPSSQSSND
bottom-up

SEP14

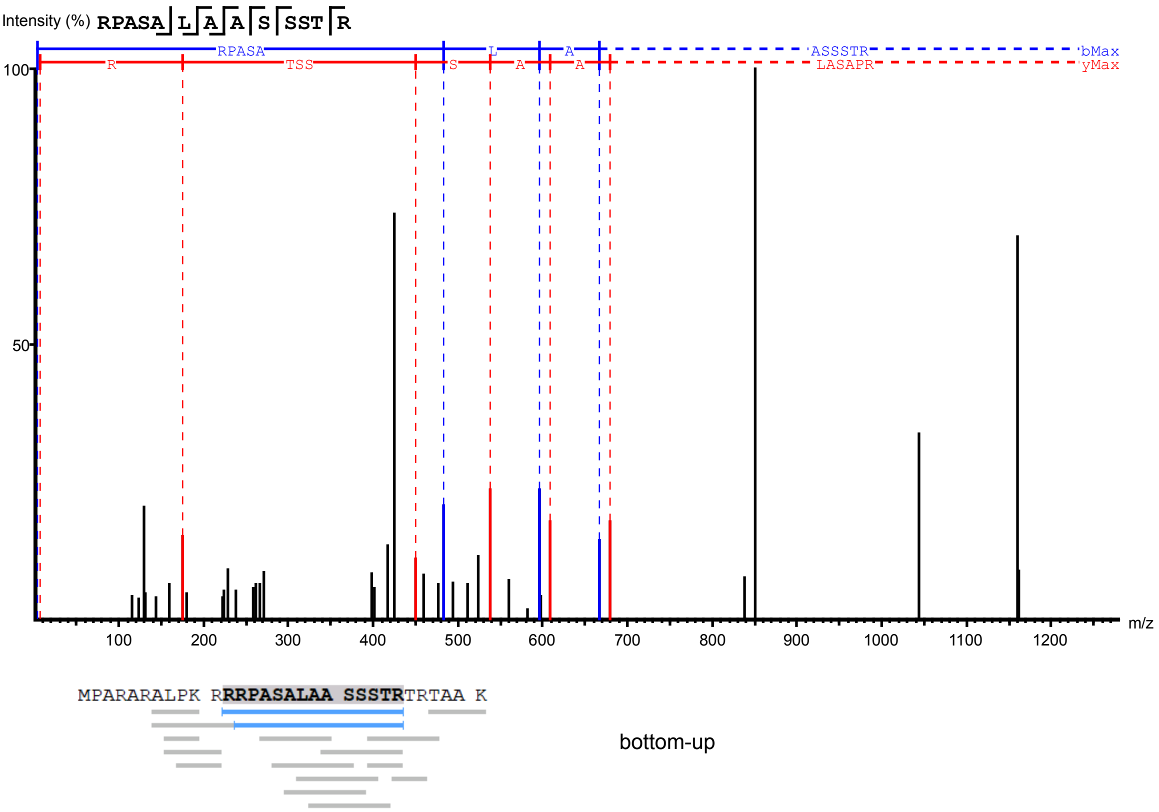

Intensity (%)
RPASA L A A S SST R
RPASA
L
A
ASSSTR
bMax
R
TSS
S
A
A
LASAPR
yMax
100
50
100
200
300
400
500
600
700
800
900
1000
1100
1200
m/z
MPARARALPK RRRPASALAA SSSTRTRTAA K
bottom-up

SEP15

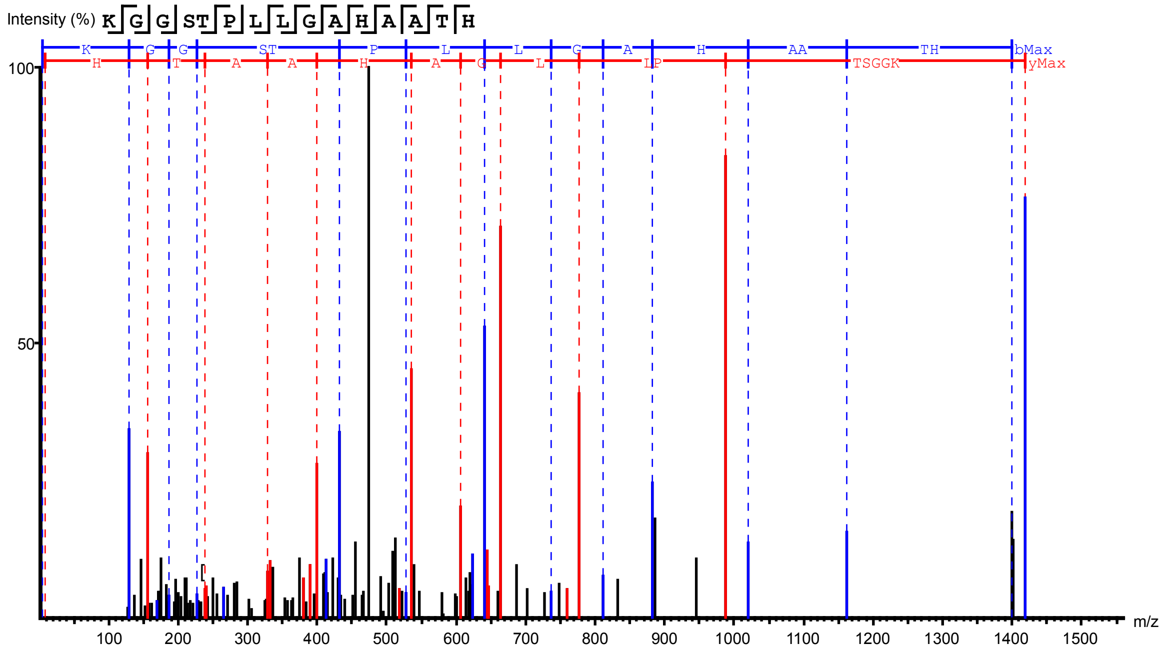
Intensity (%)
K G G ST P L L G A H A A T H
m/z
bMax
yMax

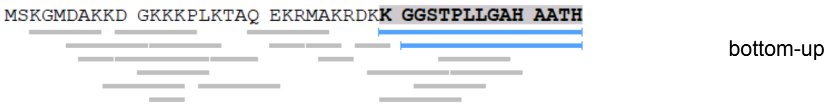
MSKGMDAKKD GKKKPLKTAQ EKRMAKRDKK GGSTPLLGAH AATH
bottom-up

SEP16

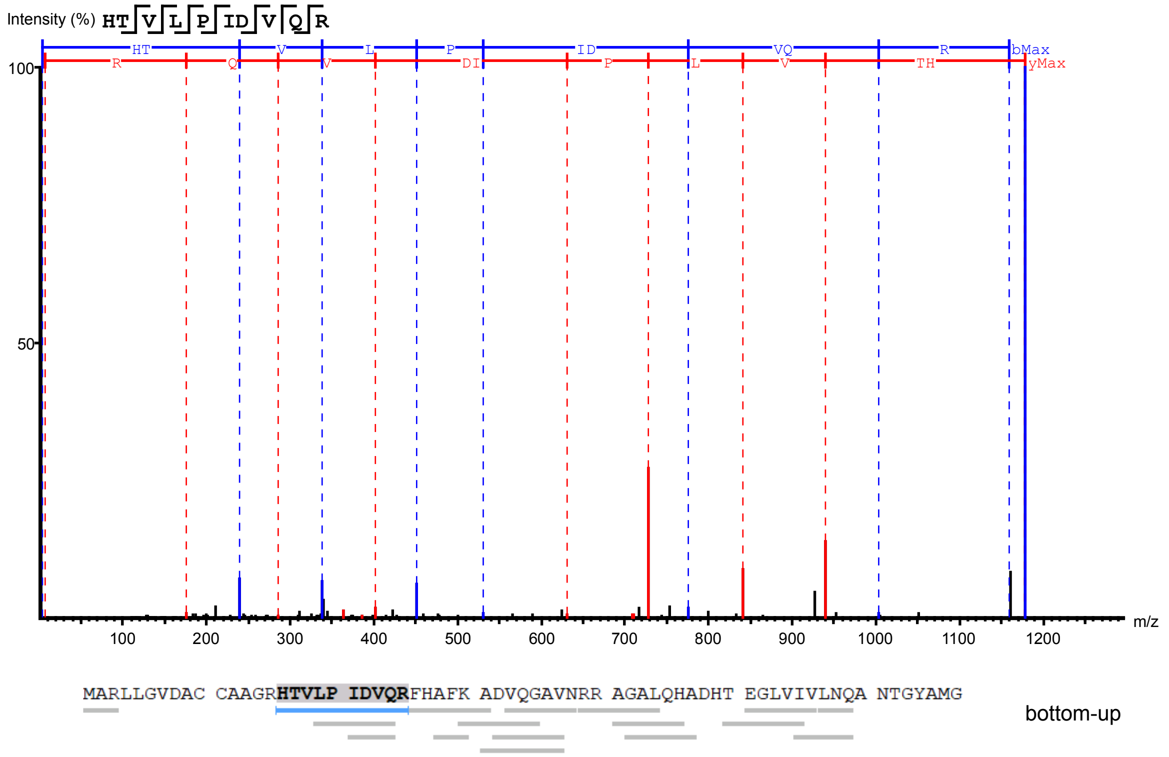

Figure S6. Example spectra and coverages for all novel small proteins (SEP) by direct sequencing and bottom-up approaches as indicated. Blue bars represent peptide matches, while grey bars show matching de novo tags.

Figure S7 A novel SEP with several possible proteoforms

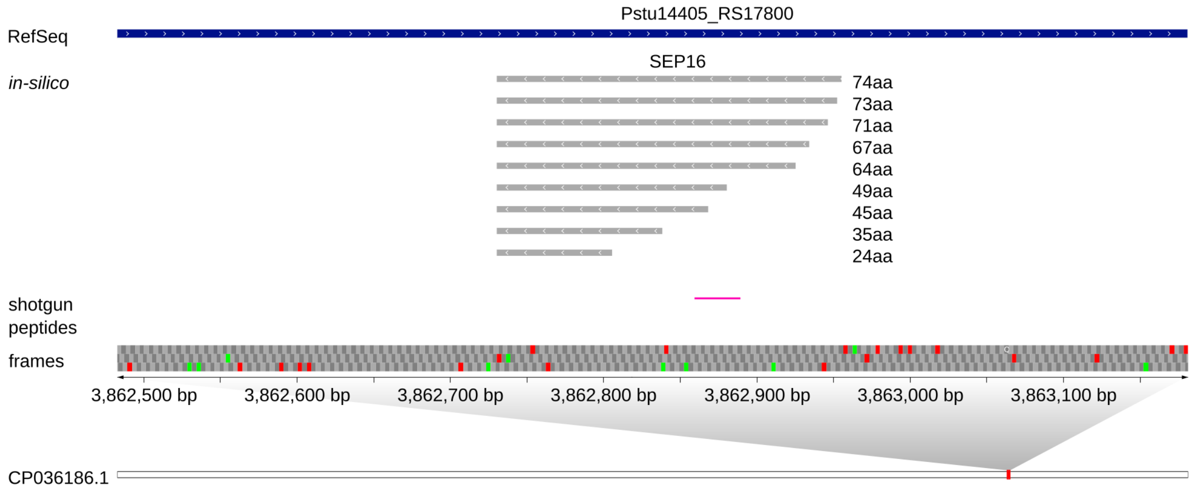

Figure S7. A novel short ORF-encoded protein (SEP) located on the opposite strand of a RefSeq annotated protein (Pstu14405_RS17800, ABC transporter permease) is implicated by a single class 2a peptide (pink)[17], that was identified by several peptide spectrum matches (PSMs) (**Table S5**). The 2a peptide evidence class indicates that the peptide identifies a subset of the possible proteoforms encoded by this annotation cluster (*in silico* prediction). The exact length of the novel SEP cannot be determined; the only detected peptide maps to five isoforms with lengths between 64 and 74 amino acids, but thereby also excludes an additional, four shorter proteoforms. For more information about peptide evidence classes and annotation clusters, please see the original description[17], the extension for proteogenomics in prokaryotes[18] and information available at the public iPtgxDB web server (https://iptgxdb.expasy.org/creating_iptgxdbs/).

Figure S8. A novel SEP encoded by multiple identical genes

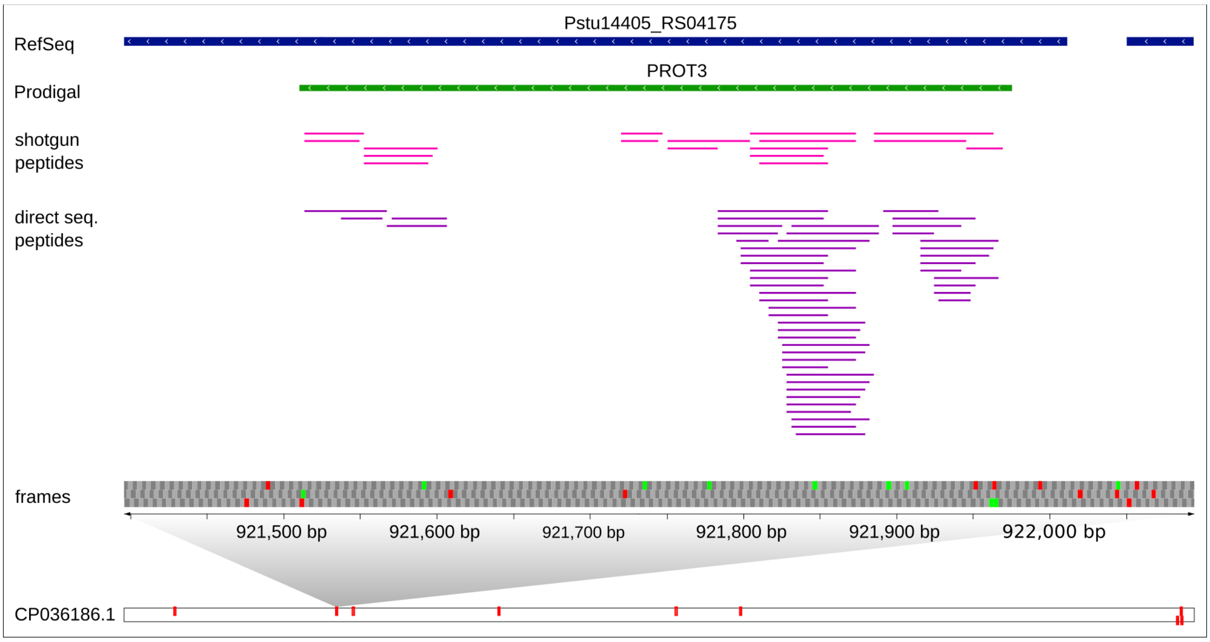

Figure S8. Peptide evidence (class 3a [17]) for a 154 aa long protein (**Table S6**) whose gene is predicted by Prodigal. This means that a protein sequence was unambiguously identified than can be encoded by several gene models (in eukaryotes, histones are examples for such duplicated, protein-coding genes). Nine exact copies of the gene exist in the *P. stutzeri* ATCC 14405 genome (their occurrences are marked as red boxes within the artificially linearized chromosome). The corresponding peptides were detected both with shotgun proteomics (17 peptides; pink) and with digest-free direct sequencing (51 peptides; purple). The Prodigal gene prediction (green) is located in-frame in a RefSeq annotated IS3-family transposase with a programmed frameshift (same for all 9 genomic locations).

Figure S9. Proline content of novel small proteins

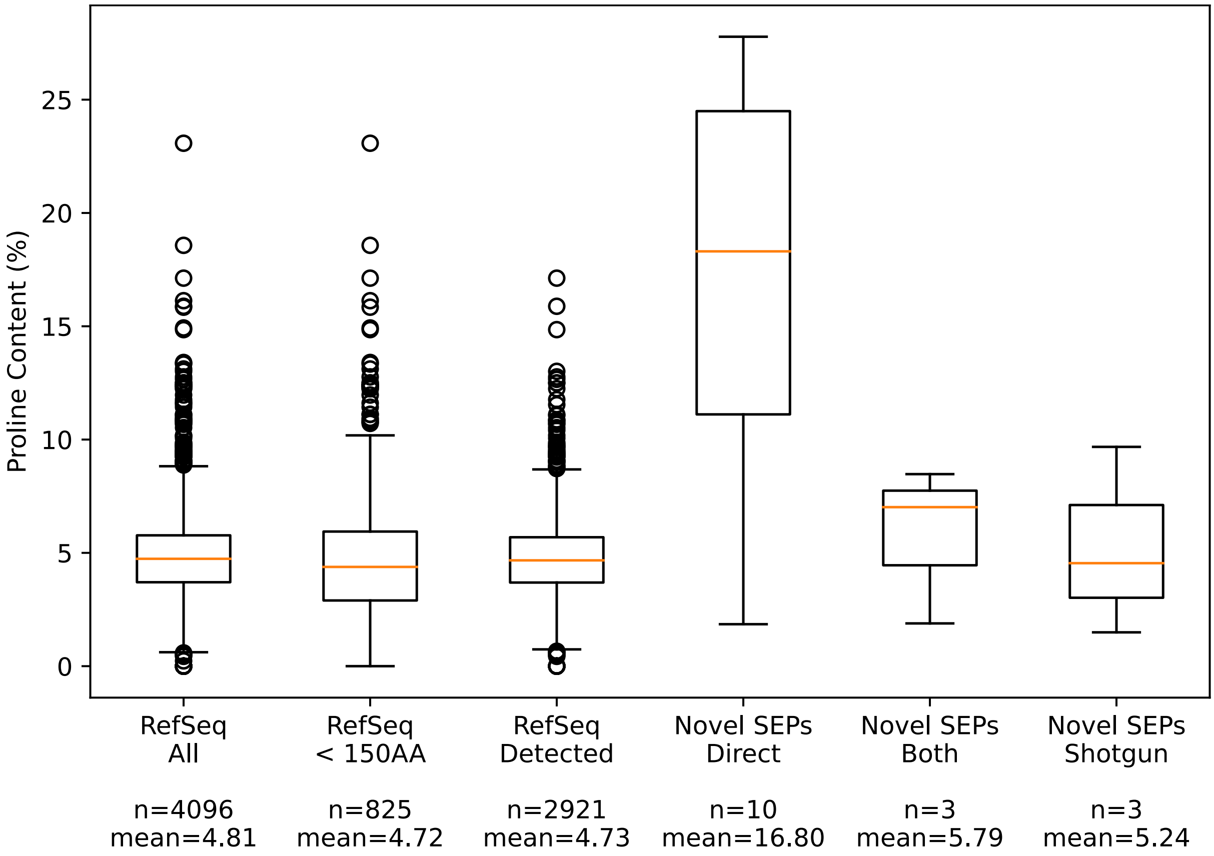

Figure S9. Proline content in different subsets of proteins (theoretical: RefSeq, RefSeq small proteins and observed in our dataset: RefSeq proteins and novel SEPs by direct sequencing, combined and shotgun-only). Direct sequencing identifies proteins with a significantly elevated proline content when compared to all other annotated proteins (Welch's t-test, p = 0.0035).

References

(1) Xie, H.; Buschmann, S.; Langer, J. D.; Ludwig, B.; Michel, H. Biochemical and biophysical characterization of the two isoforms of cbb3-type cytochrome c oxidase from *Pseudomonas stutzeri*. *Journal of Bacteriology* **2014**, *196* (2), 472-482. DOI: 10.1128/JB.01072-13.
(2) Kolmogorov, M.; Yuan, J.; Lin, Y.; Pevzner, P. A. Assembly of long, error-prone reads using repeat graphs. *Nature Biotechnology* **2019**, *37* (5), 540-546. DOI: 10.1038/s41587-019-0072-8.
(3) Li, H. Aligning sequence reads, clone sequences and assembly contigs with BWA-MEM. *arXiv preprint arXiv:1303.3997* **2013**.
(4) Garrison, E.; Marth, G. Haplotype-based variant detection from short-read sequencing. *arXiv preprint arXiv:1207.3907* **2012**.
(5) Sović, I.; Šikić, M.; Wilm, A.; Fenlon, S. N.; Chen, S.; Nagarajan, N. Fast and sensitive mapping of nanopore sequencing reads with GraphMap. *Nature Communications* **2016**, *7* (1), 11307. DOI: 10.1038/ncomms11307.
(6) Sedlazeck, F. J.; Rescheneder, P.; Smolka, M.; Fang, H.; Nattestad, M.; Von Haeseler, A.; Schatz, M. C. Accurate detection of complex structural variations using single-molecule sequencing. *Nature Methods* **2018**, *15* (6), 461-468. DOI: 10.1038/s41592-018-0001-7.
(7) Okonechnikov, K.; Conesa, A.; García-Alcalde, F. Qualimap 2: advanced multi-sample quality control for high-throughput sequencing data. *Bioinformatics* **2016**, *32* (2), 292-294. DOI: 10.1093/bioinformatics/btv566.
(8) Thorvaldsdóttir, H.; Robinson, J. T.; Mesirov, J. P. Integrative Genomics Viewer (IGV): high-performance genomics data visualization and exploration. *Brief Bioinform* **2013**, *14* (2), 178-192. DOI: 10.1093/bib/bbs017.
(9) Antipov, D.; Hartwick, N.; Shen, M.; Raiko, M.; Lapidus, A.; Pevzner, P. A. plasmidSPAdes: assembling plasmids from whole genome sequencing data. *Bioinformatics* **2016**, *32* (22), 3380-3387. DOI: 10.1093/bioinformatics/btw493.
(10) Quinlan, A. R.; Hall, I. M. BEDTools: a flexible suite of utilities for comparing genomic features. *Bioinformatics* **2010**, *26* (6), 841-842. DOI: 10.1093/bioinformatics/btq033.
(11) Krzywinski, M.; Schein, J.; Birol, I.; Connors, J.; Gascoyne, R.; Horsman, D.; Jones, S. J.; Marra, M. A. Circos: an information aesthetic for comparative genomics. *Genome Res* **2009**, *19* (9), 1639-1645. DOI: 10.1101/gr.092759.109.
(12) Meier, F.; Geyer, P. E.; Winter, S. V.; Cox, J.; Mann, M. BoxCar acquisition method enables single-shot proteomics at a depth of 10,000 proteins in 100 minutes. *Nature Methods* **2018**, *15* (6), 440-448. DOI: 10.1038/s41592-018-0003-5.
(13) Huerta-Cepas, J.; Forslund, K.; Coelho, L. P.; Szklarczyk, D.; Jensen, L. J.; von Mering, C.; Bork, P. Fast Genome-Wide Functional Annotation through Orthology Assignment by eggNOG-Mapper. *Molecular Biology and Evolution* **2017**, *34* (8), 2115-2122. DOI: 10.1093/molbev/msx148.
(14) Cock, P. J.; Antao, T.; Chang, J. T.; Chapman, B. A.; Cox, C. J.; Dalke, A.; Friedberg, I.; Hamelryck, T.; Kauff, F.; Wilczynski, B.; et al. Biopython: freely available Python tools for computational molecular biology and bioinformatics. *Bioinformatics* **2009**, *25* (11), 1422-1423. DOI: 10.1093/bioinformatics/btp163.
(15) Hunter, J. D. Matplotlib: A 2D Graphics Environment. *Computing in Science & Engineering* **2007**, *9* (3), 90-95. DOI: 10.1109/MCSE.2007.55.
(16) Omasits, U.; Quebatte, M.; Stekhoven, D. J.; Fortes, C.; Roschitzki, B.; Robinson, M. D.; Dehio, C.; Ahrens, C. H. Directed shotgun proteomics guided by saturated RNA-seq identifies a complete expressed prokaryotic proteome. *Genome Research* **2013**, *23* (11), 1916-1927. DOI: 10.1101/gr.151035.112.
(17) Qeli, E.; Ahrens, C. H. PeptideClassifier for protein inference and targeted quantitative proteomics. *Nature Biotechnology* **2010**, *28* (7), 647-650. DOI: 10.1038/nbt0710-647.
(18) Omasits, U.; Varadarajan, A. R.; Schmid, M.; Goetze, S.; Melidis, D.; Bourqui, M.; Nikolayeva, O.; Québatte, M.; Patrignani, A.; Dehio, C. An integrative strategy to identify the

entire protein coding potential of prokaryotic genomes by proteogenomics. *Genome Research* **2017**, *27* (12), 2083-2095. DOI: 10.1101/gr.218255.116.
